# Diffuse microglial responses and persistent EEG changes correlate with poor neurological outcome in a model of subarachnoid hemorrhage

**DOI:** 10.1038/s41598-024-64631-2

**Published:** 2024-06-13

**Authors:** Joseph R. Geraghty, Mitchell Butler, Biswajit Maharathi, Alexander J. Tate, Tyler J. Lung, Giri Balasubramanian, Fernando D. Testai, Jeffrey A. Loeb

**Affiliations:** 1 Present Address: Department of Neurology, University of Pennsylvania Perelman School of Medicine, 3400 Spruce St, Philadelphia, PA 19104 USA; 2 Department of Neurology & Rehabilitation, University of Illinois College of Medicine, 912 S Wood St, NPI Suite 174N, Chicago, IL 60612 USA; 3 Richard and Loan Hill Department of Biomedical Engineering, University of Illinois at Chicago, 851 S Morgan St, Chicago, IL 60607 USA; 4 Present Address: Neuroscience Doctoral Program, Medical College of Wisconsin, Suite H2200, 8701 Watertown Plank Rd, Milwaukee, WI 53226 USA; 5 Department of Neurology and Rehabilitation, University of Illinois at Chicago, NPI North Bldg., Room 657, M/C 796, 912 S. Wood Street, Chicago, IL 60612 USA; 6 Present Address: The Ohio State University School of Medicine, 1645 Neil Ave, Columbus, OH 43210 USA

**Keywords:** Subarachnoid hemorrhage, Electroencephalography, Spectral analysis, Inflammation, Microglia, Fingolimod, Diseases of the nervous system, Stroke, Stroke, Neurological disorders, Neurovascular disorders

## Abstract

The mechanism by which subarachnoid hemorrhage (SAH) leads to chronic neurologic deficits is unclear. One possibility is that blood activates microglia to drive inflammation that leads to synaptic loss and impaired brain function. Using the endovascular perforation model of SAH in rats, we investigated short-term effects on microglia together with long-term effects on EEG and neurologic function for up to 3 months. Within the first week, microglia were increased both at the site of injury and diffusely across the cortex (2.5-fold increase in SAH compared to controls, p = 0.012). Concomitantly, EEGs from SAH animals showed focal increases in slow wave activity and diffuse reduction in fast activity. When expressed as a fast-slow spectral ratio, there were significant interactions between group and time (*p* < 0.001) with less ipsilateral recovery over time. EEG changes were most pronounced during the first week and correlated with neurobehavioral impairment. In vitro, the blood product hemin was sufficient to increase microglia phagocytosis nearly six-fold (*p* = 0.032). Immunomodulatory treatment with fingolimod after SAH reduced microglia, improved neurological function, and increased survival. These findings, which parallel many of the EEG changes seen in patients, suggest that targeting neuroinflammation could reduce long-term neurologic dysfunction following SAH.

## Introduction

SAH is a devastating neurological emergency with considerable morbidity and mortality. It accounts for up to 5% of strokes each year, with 7.2–10.5 per 100,000 people affected annually^1–3^. One of the initial consequences of SAH is early brain injury (EBI) occurring within the first 72 h^4,5^. During this period, neuroinflammation and potential excitotoxic effects of blood and its breakdown products on neurons and glia could play a meaningful role in neurologic outcome^5–7^. Furthermore, events that occur acutely after SAH likely set the stage for the development of delayed brain injury and long-term disabilities.

EEG abnormalities are frequent after aneurysmal SAH. The growing use of continuous EEG in intensive care enables real-time monitoring of post-SAH brain activity^8^. SAH patient brains often exhibit increased slow waves, both locally in injured areas and throughout the entire brain. Additionally, SAH patients show reduced power in faster frequency bands, as measured by alpha-delta ratio (ADR) and relative alpha variability (RAV)^9,10^. Studies suggest that changes in ADR and RAV can help predict cerebral vasospasm and delayed cerebral ischemia (DCI), which are common complications of SAH occurring typically within 3–14 days after injury^8,10–13^.

Following the lack of success in various major clinical trials that aimed to prevent DCI after SAH, focus has shifted towards targeting earlier processes such as neuroinflammation. Microglia, the resident immune cells of the central nervous system (CNS), are the initial responders to brain injury. They play a pivotal role in triggering subsequent immune and inflammatory responses, as they secrete cytokines and chemokines that facilitate the infiltration of peripheral immune cells^6,7^. Microglia eliminate cellular debris and toxic blood products following injury and also play a role in altering synaptic networks that result in impaired neurological function^14^. Most studies of post-SAH microglial responses have investigated select neuroanatomical areas, limiting our understanding of how these cells respond throughout the entire brain^15^. Here, we mapped the spatiotemporal distribution of microglia throughout the entire SAH rodent brain with and without treatment with the immunomodulatory drug fingolimod (FTY720, hereafter referred to as FTY). We previously demonstrated the therapeutic potential of this drug, a lipophilic analog of sphingosine-1-phosphate currently used in the treatment of multiple sclerosis^16–18^, in a SAH rat model^19,20^. Therefore, given our prior results, we investigated the effects of FTY on spatiotemporal microglial changes in this study.

Given the lack of long-term studies in preclinical models of SAH, we optimized the endovascular perforation model for long-term EEG and neurobehavioral investigations in rats. MRI was performed prior to EEG studies to measure the extent and location of hemorrhage. Consistent with observations in SAH patients, our findings revealed heightened slow wave activity and concurrent reductions in fast wave activity in SAH animals. Changes in EEG were correlated with neurobehavioral abnormalities which were in turn associated with widespread microglial activation throughout the brain, even at sites considerably remote from the SAH injury within one-week post-injury. Finally, we demonstrated that treatment with FTY mitigated microglial activation and improved neurologic outcome.

## Methods

### Animals

All animal studies were approved by the University of Illinois at Chicago Office of Animal Care and Institutional Biosafety Animal Care Committee (18–110, 19–210) and conducted in accordance with the institutional and ARRIVE guidelines and regulations. Male Sprague–Dawley rats (2–4 months) were purchased from Envigo laboratory. Studies in rats were conducted in two experiments: (1) To determine long-term (up to 3 months) quantitative EEG and neurobehavioral changes after experimental SAH, and (2) To determine acute (2 days) and delayed (7 days) microglial responses following SAH. For histological studies, 4–6 animals per group were used, consistent with previous studies^20^. Given that this was the first study to evaluate long-term EEG changes in an animal model of SAH, we were unable to estimate effect sizes but aimed for a total of ten animals in our experimental group. All animals were randomized to control, disease, and treatment groups. We also performed an additional in vitro phagocytosis assay of primary microglia using neonatal pups from breeding pairs (2 months) of C57BL/6J mice under a CX_3_CR-1^GFP/GFP^ genetic background purchased from Jackson Laboratories, Inc. All animals were housed under a 12-h light–dark cycle with ad libitum access to food and water. See Supplementary Methods for further details.

### Endovascular perforation model

The endovascular perforation (EVP) model is a non-craniotomy model that recapitulates aneurysmal SAH via direct perforation of an intracranial artery, establishing a hemorrhagic lesion under arterial pressure^21–23^. Briefly, rats are first anesthetized with isoflurane in 100% oxygen, intubated, and placed on a ventilator. An incision is made over the midline scalp and a Doppler probe is attached to the skull over the territory of the right middle cerebral artery (MCA). Regional cerebral blood flow (rCBF) is continuously monitored before, during, and after surgery up to 30 min by Laser Doppler flowmetry (Perimed Inc.).

**Figure 1:**
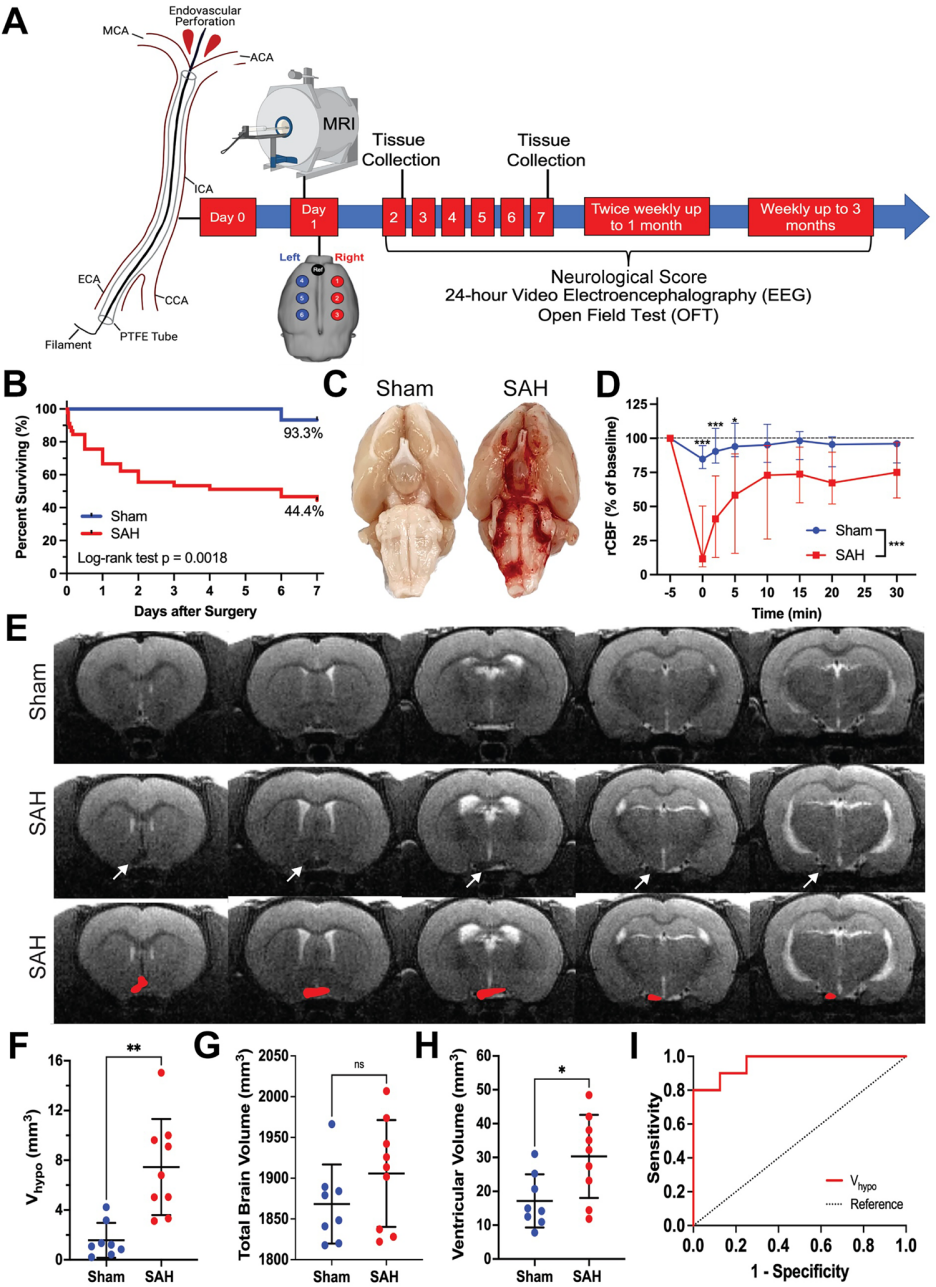
Experimental design and MRI-based characterization of an experimental SAH model optimized for long-term study. (**A**) SAH was induced at day 0. MRI was performed 24 h after surgery to assess the extent and location of bleeding, followed by implantation of epidural EEG electrodes covering the convexity of the brain. Neurologic severity scoring, 24-h video EEG, and open field testing were then conducted at regular time points up to 3 months. In a separate cohort, brains were harvested at 2 and 7 days after injury to assess for degree of inflammation. Figure created with BioRender.com. (**B**) Kaplan–Meier survival curves are shown for sham and SAH animals for up to 7 days after surgery. Log-rank (Mantel-Cox) test, *p* = 0.002. Percentages represent the proportion of animals surviving by day 7. (**C**) Direct visualization of the ventral surface of the rat brain serves as the gold standard for confirming SAH. (**D**) Laser Doppler flowmetry recordings were performed over the right middle cerebral artery territory to collect regional CBF. Baseline values were obtained pre-operatively and monitored for 30 min post-operatively. A two-way ANOVA with repeated measures showed main effects of time, group, as well as an interaction between group and time (each *p* < 0.001). *n* = 14–18 per group. Data shown as median (IQR). **E)** SAH was identified on T2-MRI as reduced hyperintense signal at the skull base corresponding to a reduction in normal cerebrospinal fluid hyperintensity. (**F**–**H**) Ventral hypointense signal volume (*V*_*hypo*_, **F**), total brain volume (**G**), and ventricular volume (**H**) were quantified in sham and SAH rats. *n* = 8–10 animals per group, independent sample t-test, data shown as mean ± standard deviation. **I**) ROC curve analysis confirmed the ability of *V*_*hypo*_ to correctly identify SAH without harvesting the brain for long-term studies. AUC = 0.963, *p* = 0.001. An optimal cutoff of *V*_*hypo*_ 4.63 mm^3^ corresponded to 80% sensitivity and 100% specificity for confirming SAH.

A second midline incision is made in the anterior neck and underlying muscles, glandular tissue, and fascia are dissected until the right common carotid artery (CCA) is visualized. The bifurcation of the CCA is identified, at which point the external carotid artery (ECA) and internal carotid artery (ICA) are dissected. The ECA is dissected anteriorly, cauterized, and divided to create a proximal ECA stump, and a small arteriotomy is made. A hollow polytetrafluoroethylene tube (SUBL-080, OD 0.2 mm, ID 0.1 mm, Braintree Scientific, Inc.) containing a tungsten wire (W91, 0.076 mm diameter, Scientific Instrument Services, Inc.) is inserted into the ECA and carefully advanced into the ICA approximately 2 cm until a drop in rCBF occurs. The tungsten wire is then advanced 2 mm within the tubing, puncturing the ICA bifurcation (Fig. 1A). The wire and tubing are removed and rCBF is monitored for 30 min. Sham animals underwent the same procedure except for advancement of the tungsten filament.

### Drug treatment

FTY was purchased from Cayman Chemicals, prepared in 0.9% sterile saline, and administered as a single dose of 0.5 mg/kg intraperitoneally three hours following surgery. This dose was chosen for two reasons: it falls within the range of doses for this drug typically used in rodents, and previous studies have demonstrated efficacy at this dose^20^. The human equivalent dose is 0.08 mg/kg. Given increased incidence of infections following SAH^24^, our goal was not to achieve complete immunosuppression, but rather to blunt the initial inflammatory response that we hypothesized to be detrimental. Therefore, we chose a single dose lower than the maximum used in rodents which would have resulted in more significant immunosuppression.

### Magnetic resonance imaging

T2-weighted MRI using an Agilent 9.4 Tesla scanner was performed 24 h after surgery under isoflurane anesthesia (Fig. 1A). SAH was identified as hypodense signal on the ventral surface of the brain near the skull base, which normally appears hyperdense due to the presence of cerebrospinal fluid (CSF) within the basal cisterns^25^. In each animal, hypodense regions of interest (ROIs) were identified by two independent, blinded observers. The average hypodense volume (*V*_*hypo*_) was calculated between the independent reviewer-identified measurements. For each animal, we also measured the total brain volume and ventricular volume.

### Electroencephalography

Epidural EEG electrodes were implanted in both sham and SAH animals following surgery. To assess location-specific changes in brain activity, six recording electrodes (three per hemisphere, L1–L3 and R1–R3) were placed over the convexity of the brain^26–28^. EEG acquisition was performed according to recently published guidelines for standardizing EEG studies in rodents^29–31^. 24-h EEG recordings (Stellate HARMONIE 6.0, Stellate Systems Inc.) were conducted at a 1000-Hz sampling rate using a LaMont Pro-36 Programmable Amplifier (Lamont Medical Instruments). All EEGs were conducted with simultaneous video using an infrared-enabled camera that recorded both light and dark cycles. Animals were able to move freely and with ample space in clear acrylic recording cages. EEG recordings were obtained from each animal at regular time points (Fig. 1A). EEG data was reviewed manually in both referential and average reference montages to assess overall quality and exclude motion artifacts (e.g., chewing, grooming) based on synchronous video. Data was then processed through custom artifact removal software. Representative raw EEG examples are shown.

### Spectral analysis

We evaluated the mean spectral power in the following frequency bands: delta (δ, 1–4 Hz), theta (*θ*, 4–8 Hz), alpha (*α*, 8–12 Hz), beta (*β*, 12–30 Hz), as well as a broadband range (1–20 Hz). All power calculations were performed in MATLAB (MathWorks Inc., Natick, MA). EEG recordings were divided into continuous, non-overlapping 5-min epochs. The power spectrum in each epoch was estimated using a short-time Fourier transform with a hamming window, and the average power was calculated by integrating the spectrum over the frequency range of interest. We measured relative frequency band variability, defined as the power in the specified frequency band divided by the broadband range power. We also calculated alpha-delta ratio (ADR = *α*/*δ*) and fast-slow spectral power ratio (SPR = *[α* + *β]*/*[δ* + *θ]*). Given published literature of clinical studies in SAH as well as the relationship of SPR to neurobehavioral impairments^9,10,32^, we specifically focused on SPR, mean delta power, RAV, and ADR. Spectral power was analyzed on each individual electrode and compared across experimental groups over time.

### Neurobehavioral assessment

To evaluate neurologic deficits, we used the modified Garcia neurological score, a 21-point scale that assesses spontaneous activity, coordination, balance, sensory reflexes, limb flexion and extension, gait, and grip strength^33–35^. Neurologic scoring was conducted prior to surgery in all animals to document baseline function and continued postoperatively after each EEG recording (Fig. 1A). We performed the open field test for 15 min to measure spontaneous locomotor activity and anxiety-like behavior^36^. Spontaneous locomotor activity was measured by ambulatory distance, vertical counts (rearing), and the ratio of counterclockwise to clockwise (CCW:CW) rotations. For anxiety-like behavior, the natural tendency of animals to avoid the center of an open field (thigmotaxis) was assayed and measured as percentage of time spent in the center^34,37^. Open field tests were conducted weekly after each 24-h EEG recording (Fig. 1A). Behavior was not performed simultaneously as EEG equipment tethering could restrict range of motion. All assessments were performed by an investigator blinded to experimental group.

### Immunohistochemistry

Rats were euthanized at 2 days, 7 days, or 3 months after surgery via isoflurane overdose followed by transcardial perfusion with ice-cold heparinized saline (10 U/mL) and 4% paraformaldehyde. Brains were harvested and post-fixation at 4 °C was performed in 4% paraformaldehyde for 16 h. Brains were transferred to a 15% sucrose solution for 24 h, followed by a 30% sucrose solution for 24 h. Brains were divided into seven 3 mm blocks along the rostral-caudal axis, embedded, frozen, and sectioned at 20 µm thickness.

Microglial immunohistochemistry was performed using a polyclonal rabbit anti-Iba-1 antibody (1:750, FUJIFILM Wako Pure Chemical Corporation, 019-019741) overnight at 4 °C. Secondary antibody staining was performed using an avidin/biotin-based peroxidase system with a biotinylated anti-rabbit IgG secondary antibody (VECTASTAIN® Elite ABC-HRP Kit, PK-61-1, Vector Laboratories, Inc.) for one hour at room temperature. To assess for the presence of blood products, Prussian Blue staining was performed to identify deposits of ferric iron (Abcam ab150674). Slides were dehydrated using an ethanol gradient (70%, 95%, 100%) and cleared with xylene, each for 4 min. Slides were imaged using a Leica Aperio AT2 whole slide brightfield scanner at 20X magnification.

To visualize changes in Iba-1^+^ cell responses throughout the entire brain, we identified 64 ROIs (32 per hemisphere) across all seven brain blocks based on well-defined coordinates using a rat brain atlas. Within each hemisphere, ROIs were selected to capture known areas of hemorrhage from MRI as well as representative samples of relevant neuroanatomical structures. Prior to ROI selection, all images were coded. ROIs were selected and manually thresholded by a blinded investigator to selected intensity that captured the entire Iba-1^+^ cell body. Percent Iba-1^+^ area and average Iba-1^+^ object size were calculated to analyze the amount and size of microglia present within each field.

### Microglial phagocytosis assay

Mixed glial cell cultures were collected from the brains of CX_3_CR-1^GFP/GFP^ C57BL/6J mouse pups (day 0–5, male and female). After 10–12 days, microglia were collected and plated for 24 h. The phagocytic activity of primary microglia in response to the blood product hemin was assayed using a fluorescent latex bead phagocytosis assay^38^. Hemin (40 µM in 133 µM NaOH) or vehicle (133 µM NaOH) was added in the presence of two-micron, fluorescent blue, modified amine polystyrene latex beads in aqueous solution (0.02% final concentration, Sigma-Aldrich, Inc.), incubated for 12 h at 37 °C in 5% CO_2_, washed, and fixed in 4% PFA for 30 min. This hemin concentration is well below the expected 10 mM concentration expected to be formed from 2.5 mM hemoglobin within erythrocytes but has been shown in previous studies to cause neurotoxicity^39,40^. All cell culture experiments were run in duplicate within an experiment, and two independent experiments were conducted to ensure reproducibility. Slides were imaged using a Leica ctr5500 scope, LS200US laser box, Qimaging exi aqua camera, and Surveyor imaging software. Two images were acquired from each well, corresponding to four images per group within each independent experiment. Images were coded and analyzed by an independent reviewer blinded to the experimental conditions. The total number of GFP^+^ cells in each field and the number of beads within each cell was counted.

### Statistical analysis

Data was assessed for normality using the Shapiro–Wilk test. Independent sample t-test was used to compare MRI quantifications between sham and SAH animals as well as bead phagocytosis between hemin- and vehicle-treated primary microglia. To compare measures of rCBF, neurologic scores, open field assessments, and Iba-1 immunohistochemistry over time and across groups, two-way ANOVA was performed. When data involved multiple time points measured from the same animal, repeated measures analysis was used. Šidák’s multiple comparisons test was used to assess for specific between-group differences. For quantitative EEG, three-way ANOVA was performed to ascertain both main effects and interactions among group, electrode, and time. Within each electrode, Šidák’s multiple comparisons test was used to assess for between-group differences at each time point. Survival data was shown as a Kaplan–Meier survival curve and analyzed using a log-rank (Mantel-Cox) test. Receiver operating characteristic (ROC) analysis was performed to assess the ability of MRI to confirm SAH compared to the gold standard of visual inspection of the brain after euthanasia. A cutoff with 100% specificity is reported. Spearman correlation test was conducted to assess relationships between continuous variables. A *p*-value of < 0.05 was considered statistically significant for all analyses unless otherwise stated (*p < 0.05, **p < 0.01, ***p < 0.001). GraphPad Prism (Version 9) and Statistical Package for the Social Sciences (Version 28, IBM® SPSS®) were used to conduct the analysis.

## Results

74 rats (15 sham, 45 SAH, and 14 SAH-FTY) were used to conduct the experiments—34 animals for long-term EEG studies and 40 for MRI and histology. We first established the model, comparing long-term differences between sham and SAH animals. Consistent with previous data seen in this model and in human SAH patients^41,42^, overall mortality across all groups was 40.5% (30/74), with the highest mortality occurring within 48 h of surgery (Fig. 1B). The overall mortality rate was 55.6% (25/45) in SAH animals compared to 6.7% (1/15) in sham animals (*p* = 0.002, Fig. 1B). No mortality was observed after 7 days in sham or SAH animals.

### In Vivo detection of SAH

The gold standard for confirming successful induction of SAH involves direct visual inspection of the ventral surface of the brain after euthanasia (Fig. 1C); however, this cannot be done in long-term studies because blood is resorbed over time^43^. Instead, to confirm SAH and measure the extent and location of bleeding, we optimized an in vivo method for detection of SAH using intraoperative rCBF over the MCA territory and MRI. At 0 min, the median drop in rCBF from baseline was 84.8% in SAH animals compared to 11.6% in shams (Fig. 1D). Over time, the rCBF showed partial recovery in SAH animals but plateaued around 10 min.

After SAH, the normally hyperintense signal at the base of the brain due to CSF on MRI became hypodense (Fig. 1E), enabling us to approximate subarachnoid blood volume (*V*_*hypo*_) in SAH animals compared to shams (7.45 ± 3.86 vs. 1.56 ± 1.40 mm^3^, *p* = 0.001, Fig. 1F). We found no difference in total brain volume between SAH and shams (1906.0 ± 65.6 vs. 1868.0 ± 48.5 mm^3^, *p* = 0.205, Fig. 1G), but ventricular volume was higher in SAH animals (30.3 ± 2.3 vs. 17.2 ± 7.9 mm^3^, *p* = 0.021, Fig. 1H). *V*_*hypo*_ successfully distinguished SAH animals from shams (AUC = 0.963, SE = 0.039, *p* = 0.001, Fig. 1I). A cutoff of 4.63 mm^3^ corresponded to an 80% sensitivity and 100% specificity to confirm SAH.

### SAH triggers increased slow and decreased fast EEG activity

**Figure 2:**
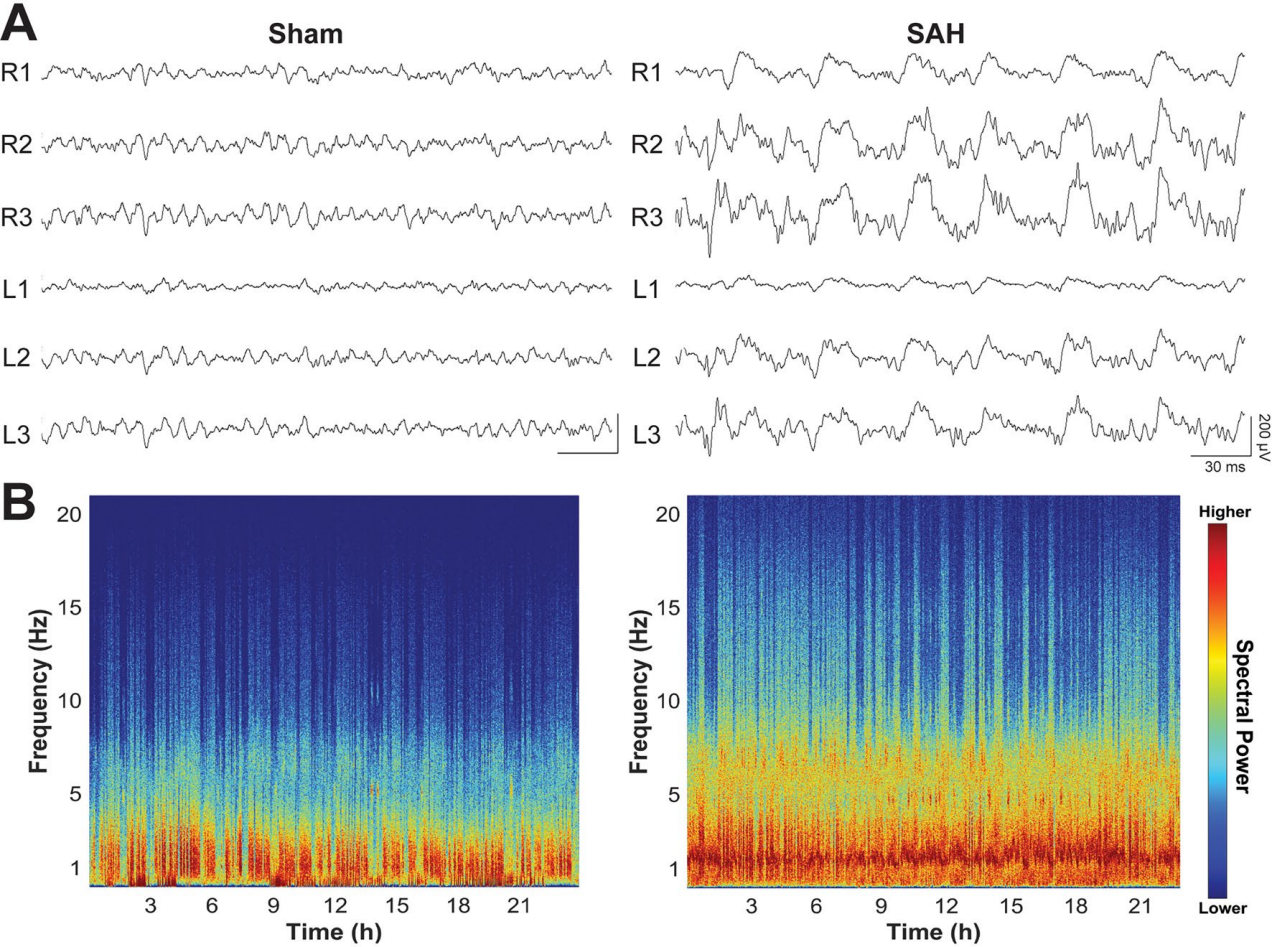
Early raw EEG changes and spectrogram after experimental SAH. (**A**) EEGs from representative sham (left) and SAH (right) animals at two days following surgery show a significant increase in slow activity throughout the brain. Data shown in referential montage, scale: 30 ms on *x*-axis, 200 µV on *y*-axis). (**B**) EEG spectrograms from full 24-h recordings are shown for the same animals 2 days following SAH. Compared to shams (left), SAH animals (right) exhibited an increase in overall spectral power that was most pronounced at lower frequencies, especially the delta band (1–4 Hz).

**Figure 3:**
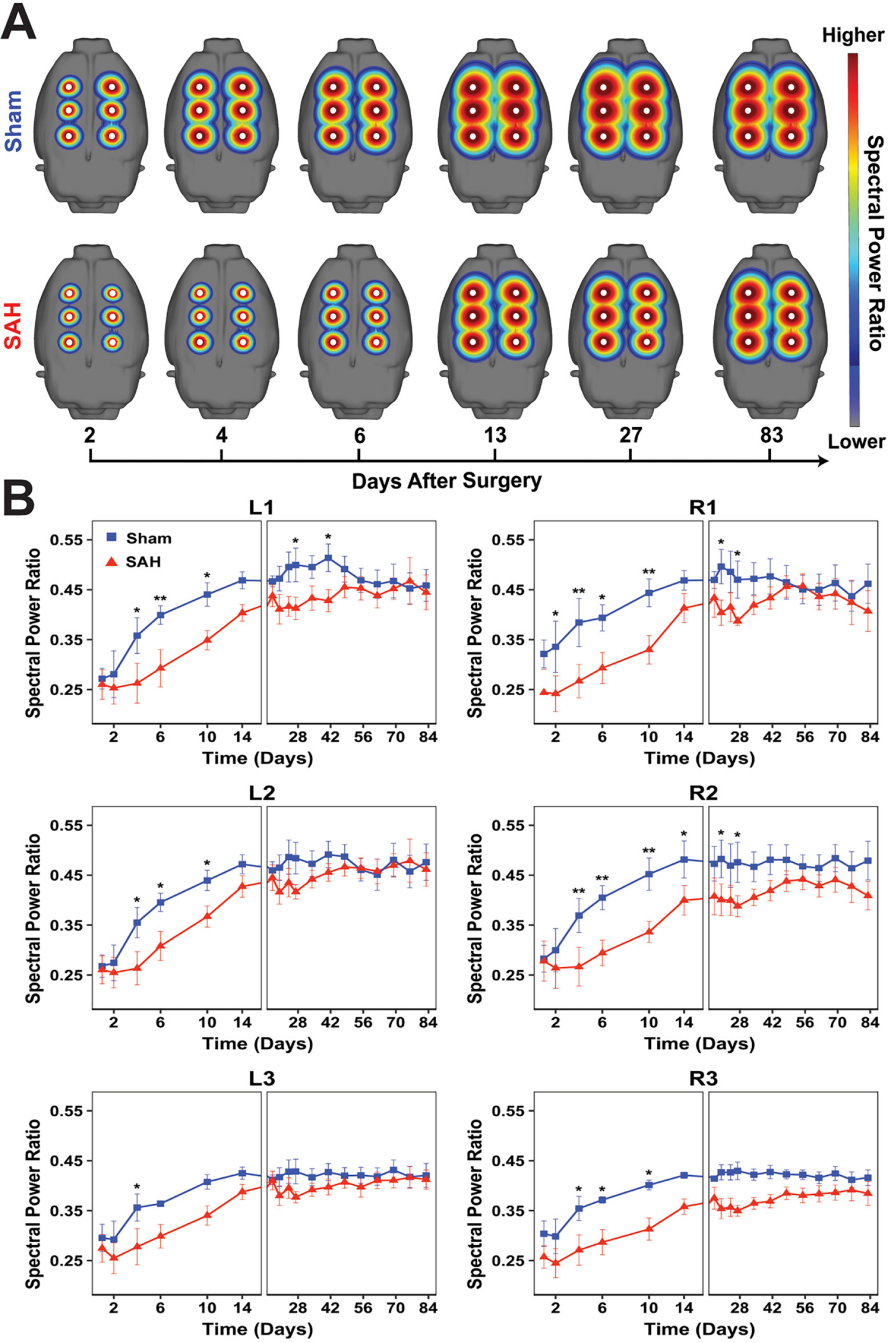
Widespread, persistent reduction in spectral power ratio after experimental SAH reflects changes in both fast and slow EEG frequency bands. (**A**) Maps of the fast-slow spectral power ratio (SPR = *[α* + *β]*/*[δ* + *θ]*) across all six electrodes are shown for sham and SAH groups over time. (**B**) Quantitative measurements showed a widespread reduction in SPR in the first 14 days after SAH across all electrodes. In some electrodes, reduced SPR persisted beyond 14 days and never recovered to baseline. Three-way ANOVA with main effects of time (*p* < 0.001), group (*p* < 0.001), electrode (*p* < 0.001) and interactions between group and time (*p* < 0.001). No interaction was detected between group and electrode (*p* = 0.062), between electrode and time (*p* > 0.999) or between all three variables (*p* > 0.999). Within each electrode, Šidák’s multiple comparisons test was used to assess for between-group differences at each time point. *n* = 6–10 animals per group. Data shown as mean ± standard error.

We next assessed longitudinal EEG changes in the SAH rodent brain. Consistent with observations in human patients, manual review of unprocessed EEG data revealed heightened slow-wave activity in SAH animals compared to shams early after injury (Fig. 2A). Given the amount of EEG data collected, we turned to quantitative EEG as a powerful technique to assess spectral changes over time. Quantitative spectral analysis showed increased power in lower delta and theta frequency bands compared to faster alpha and beta bands (Fig. 2B). SAH animals showed reduced SPR compared to shams, and this was most pronounced in the first week after injury (Fig. 3A). When analyzing group differences over time per electrode, SAH animals had lower SPR across all electrodes, mostly within the first 1–2 weeks following injury (Fig. 3B). SAH animals showed recovery of SPR over time, but this never reached the same level as shams in the R2 and R3 electrodes that typically overlap with the largest area of subarachnoid blood.

**Figure 4:**
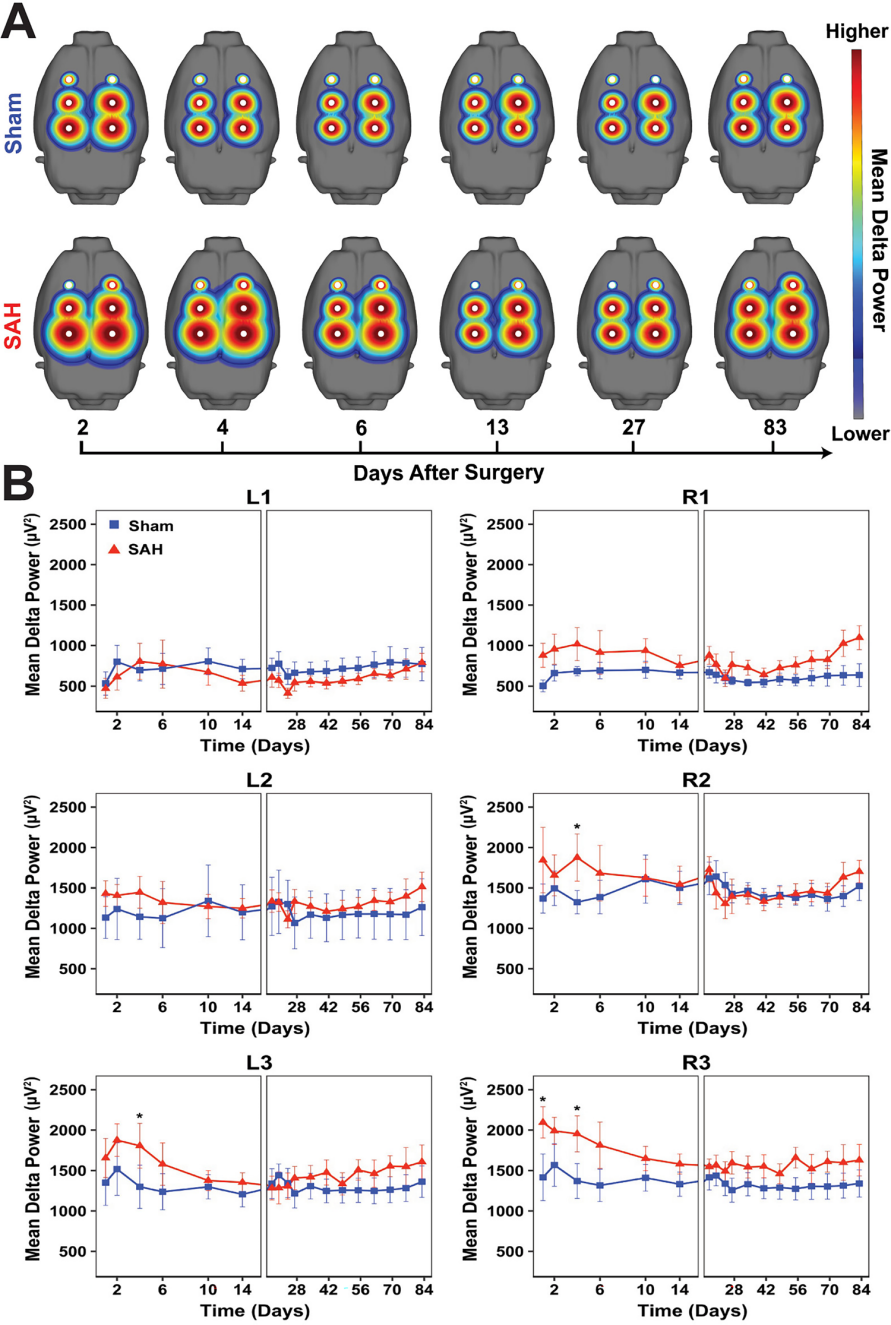
Increased delta power localizes to the site of hemorrhage early after experimental SAH. (**A**) Spectral maps of absolute delta power (1–4 Hz) across all six electrodes are shown for sham and SAH groups over time. (**B**) Quantitative EEG revealed increased delta power that mapped to hemorrhage areas, specifically the ipsilateral R2 and R3 and contralateral L3 electrodes which are closest to the regions overlying the bifurcation of the right internal carotid artery. Three-way ANOVA with main effects of time (*p* = 0.027), group (*p* < 0.001), electrode (*p* < 0.001) and interactions between group and electrode (*p* < 0.001). No interaction was detected between group and time (*p* = 0.175), electrode and time (*p* > 0.999), or between all three variables (*p* > 0.999). Within each electrode, Šidák’s multiple comparisons test was used to assess for between-group differences at each time point. *n* = 6–10 animals per group. Data shown as mean ± standard error.

While SPR is a useful metric that encapsulates changes in multiple frequency bands, we further investigated changes in specific absolute and relative EEG frequency bands (Supplementary Table 1), with close attention to those that have previously been described in SAH patients^9,10^. Compared to shams, SAH animals showed increased delta power (Fig. 4A), which mapped to areas of hemorrhage, specifically the ipsilateral R2 and R3 and contralateral L3 electrode (Fig. 4B). Significant per-electrode differences in delta power were observed on R2 at day 4 (*p* = 0.026), R3 at days 1 (*p* = 0.026) and 4 (*p* = 0.019), and L3 at day 4 (*p* = 0.042), suggesting that increased delta activity in SAH occurs predominantly within the acute period. Together, these results suggest that shortly after experimental SAH there is increased slowing which maps spatially to sites closest to the area of hemorrhage.

RAV showed a global reduction visible across all electrodes in SAH animals compared to shams (Supplementary Fig. 1). Like SPR, RAV was decreased throughout all electrodes predominantly during the first week after injury. Similar but weaker differences were observed with ADR, which was decreased in SAH animals across several electrodes (Supplementary Fig. 2). Pairwise analysis comparing sham and SAH animals over time on a per-electrode basis revealed significant differences mainly in the anterior L1, R1, and R2 electrodes more distant from the site of hemorrhage. Collectively, decreased RAV and ADR observed in SAH animals recapitulate observations in SAH patients and reflect a transition of normal brain activity to an abnormal state characterized by increased slow waves and decreased fast alpha activity relative to other frequency bands^10,44^. These data suggest that spectral EEG changes observed in SAH patients can be recapitulated in an animal model, and that certain frequency bands are affected globally while others localize to the site of injury.

### Neurobehavioral deficits correlate with EEG changes after SAH

**Figure 5:**
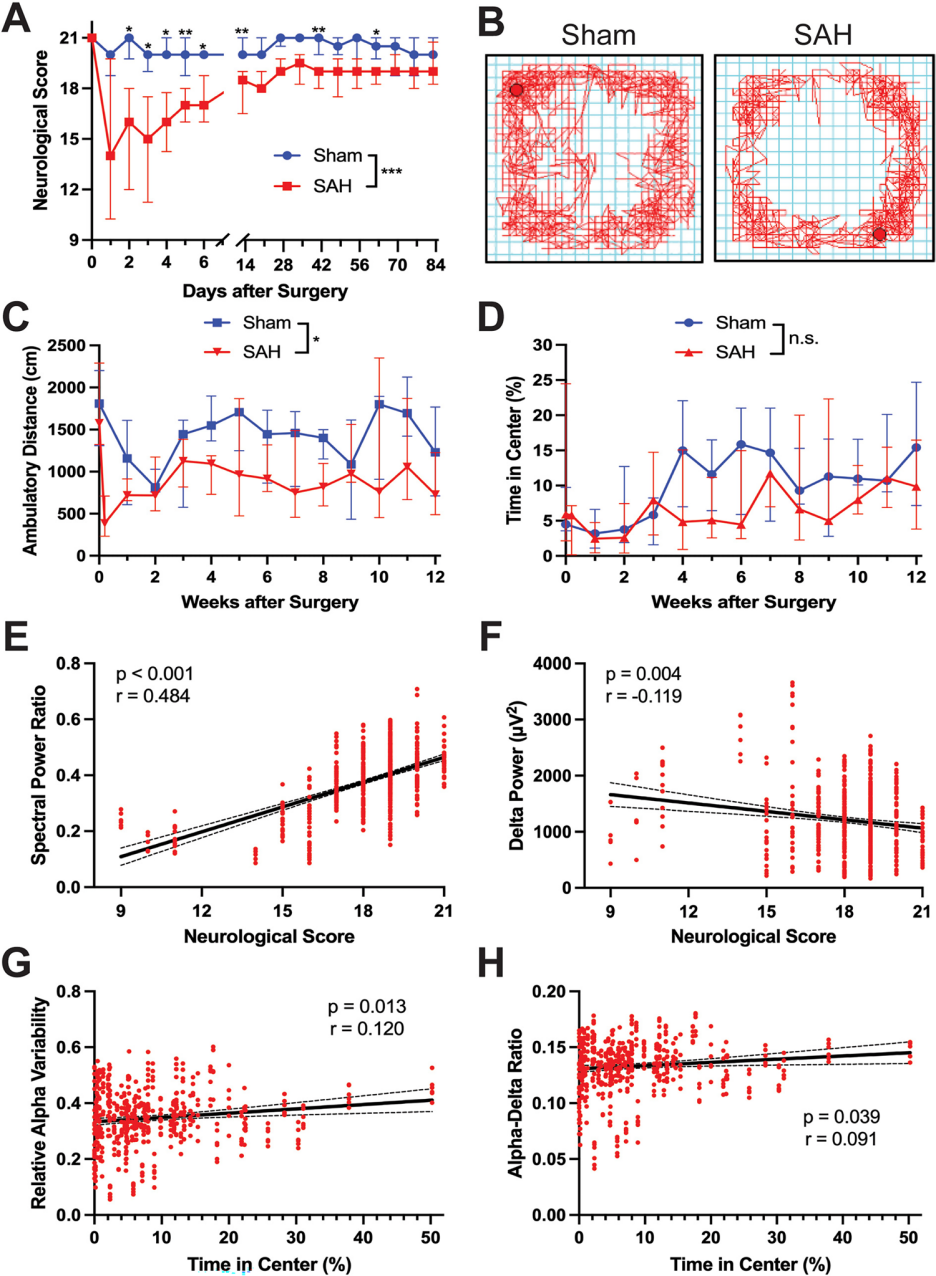
SAH produces neurobehavioral deficits that correlate with changes in EEG spectral activity. (**A**) The 21-point modified Garcia score was performed in sham and SAH animals regularly up to 12 weeks. Deficits in SAH animals were clear early on and persisted over time (two-way ANOVA: time *p* < 0.001, Sham vs. SAH *p* < 0.001, interaction *p* < 0.001). (**B**) Representative raw data from the open field test demonstrated reduced locomotor activity in SAH animals. (**C**) SAH animals showed decreased ambulatory distance compared to shams. (**D**) No differences were seen between sham and SAH animals in the percentage of time spent in the center of the open field apparatus (*n* = 6–10 animals pr group. Data shown as median ± IQR). (**E**) Spectral power ratio (SPR = *[α* + *β]*/*[δ* + *θ]*) showed a positive correlation with neurological score (Spearman correlation: *p* < 0.001, *r* = 0.484). (**F**) Absolute delta power and neurological score were inversely correlated (Spearman correlation: *p* = 0.004 *r* = − 0.119. (**G**,**H**) and positive correlations were observed between (**G**) relative alpha variability (RAV = *α* / broadband, *p* = 0.013, *r* = 0.120) and (**H**) alpha-delta ratio (ADR = *α / δ*, *p* = 0.039, *r* = 0.091). Individual data points shown were overlaid with a linear regression line and 95% confidence interval.

By 24 h, SAH animals showed a sharp decrease in neurologic function compared to shams and while some showed partial recovery by 10 weeks after injury, most never reached the level of shams (Fig. 5A). Median neurologic score at 24 h was 14/21 in SAH animals compared to 20/21 in shams. On the open field test (Fig. 5B), SAH animals displayed persistently decreased ambulatory distance (*p* = 0.041, Fig. 5C) and vertical counts (*p* = 0.026, Supplementary Fig. 3A) compared to shams. There was no group difference between sham and SAH animals in time spent in center (*p* = 0.362, Fig. 5D) or CCW:CW ratio (*p* = 0.328, Supplementary Fig. 3B).

We next examined the relationship between neurobehavioral decline and EEG alterations in SAH animals. Significant correlations were observed between neurological score and various EEG parameters, including SPR (*p* < 0.001, *r* = 0.484, Fig. 5E), delta power (*p* = 0.004, *r* = − 0.119, Fig. 5F), RAV (*p* < 0.001, *r* = 0.462, Supplementary Fig. 3C), and ADR (*p* < 0.001, *r* = 0.475, Supplementary Fig. 3D). SPR showed the strongest correlation where SAH animals with higher neurological scores had increased SPR (Fig. 5E). Consistent with increased slow-wave activity indicating general neurophysiological disturbance, we observed a weak inverse correlation between neurological score and delta power (Fig. 5F). No significant correlations were observed between ambulatory distance and EEG changes (all *p* > 0.05, Supplementary Fig. 3E–H). However, correlations were observed between time spent in the center of the open field apparatus and both RAV (*p* = 0.013, *r* = 0.120, Fig. 5G) and ADR (*p* = 0.039, *r* = 0.091, Fig. 5H). There were no significant differences between time in center and either delta power or SPR (*p* < 0.05, Supplementary Fig. 3I,J). These results identify a clear association between EEG changes after SAH and long-term neurobehavioral deficits in this model.

### SAH triggers diffusely increased microglia that is attenuated by FTY

Given that synaptic rewiring by microglia might account for the EEG and neurobehavioral changes observed in this model, we quantified brain-wide microglial responses after SAH. Since EEG changes were most prominent in the first week after SAH, we focused on early time points at post-bleed days 2 and 7. We have previously shown that the immunomodulatory drug FTY can improve neurological outcomes after SAH; therefore, a third group of FTY-treated SAH rats was included^20^. FTY-treated SAH animals showed a similar drop from baseline in rCBF to untreated SAH animals (Supplementary Fig. 4A) and a similar volume of subarachnoid blood between SAH and SAH-FTY animals (Supplementary Fig. 4B). The treatment did not change either the total brain (Supplementary Fig. 4C) or ventricular volume (Supplementary Fig. 4D).

**Figure 6:**
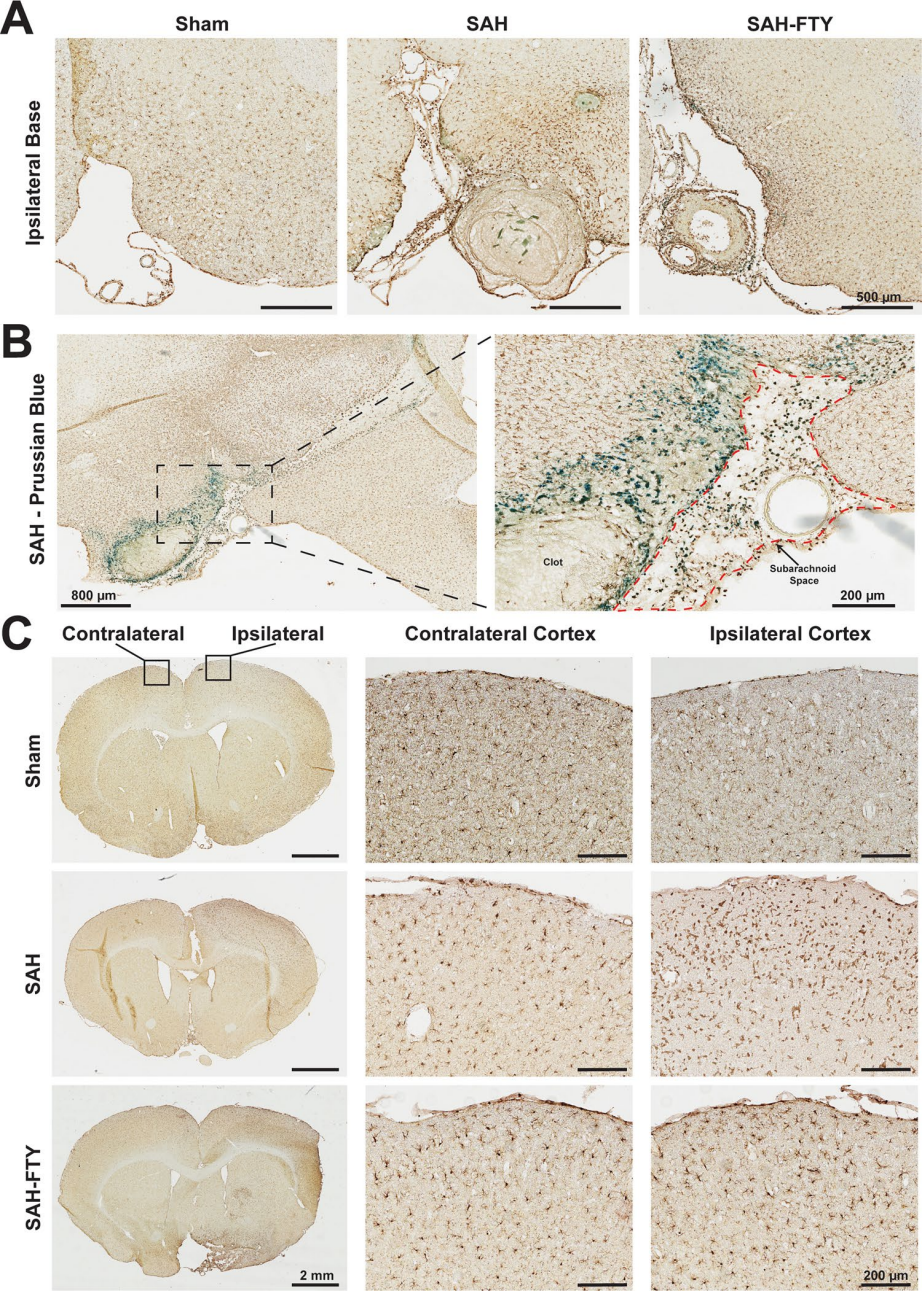
SAH results in widespread microglial responses most pronounced near the site of hemorrhage. (**A**) Iba-1 immunohistochemistry in sham and SAH animals 2 days after surgery demonstrated increased Iba-1^+^ area in untreated SAH and SAH-FTY animals compared to shams that was more pronounced ipsilateral to the injury site at the base of the brain. In addition to increased parenchymal Iba-1^+^ staining, clots were observed in the subarachnoid space with surrounding Iba-1^+^ cells. (**B**) Iba-1 and Prussian Blue co-stain reveal co-localization of Iba-1^+^ cells with blood products both within the subarachnoid space (right inset, red outline) and the nearby brain parenchyma at the ipsilateral base of the brain. (**C**) Increased Iba-1^+^ area was also observed distant from the site of hemorrhage in the cortex, particularly on the side ipsilateral to injury in untreated SAH and SAH-FTY animals compared to sham controls.

We next performed qualitative examination of serial sections throughout the brain stained for Iba-1^+^ microglia and Prussian Blue for blood products. Increased microglia were observed not only near the site of perforation and subarachnoid blood, but globally throughout the SAH brain (Fig. 6A). Many of these cells also stained positively for Prussian Blue (Fig. 6B), suggesting microglial phagocytosis of extracellular blood products. While most of the Prussian blue stain was observed in the subarachnoid space, there was evidence of Iba-1-Prussian blue co-localization within the brain parenchyma as well. Similar observations of increased microglia were seen in SAH-FTY animals at 2 days after injury (Fig. 6A). Given our findings that SAH results in concurrent cortical EEG changes and neurobehavioral impairment, we specifically assessed for Iba-1^+^ cells along the ipsilateral and contralateral cortex (Fig. 6C). Compared to shams, SAH animals had increased Iba-1^+^ cells within the cortex, most prominent within the ipsilateral cortex.

**Figure 7:**
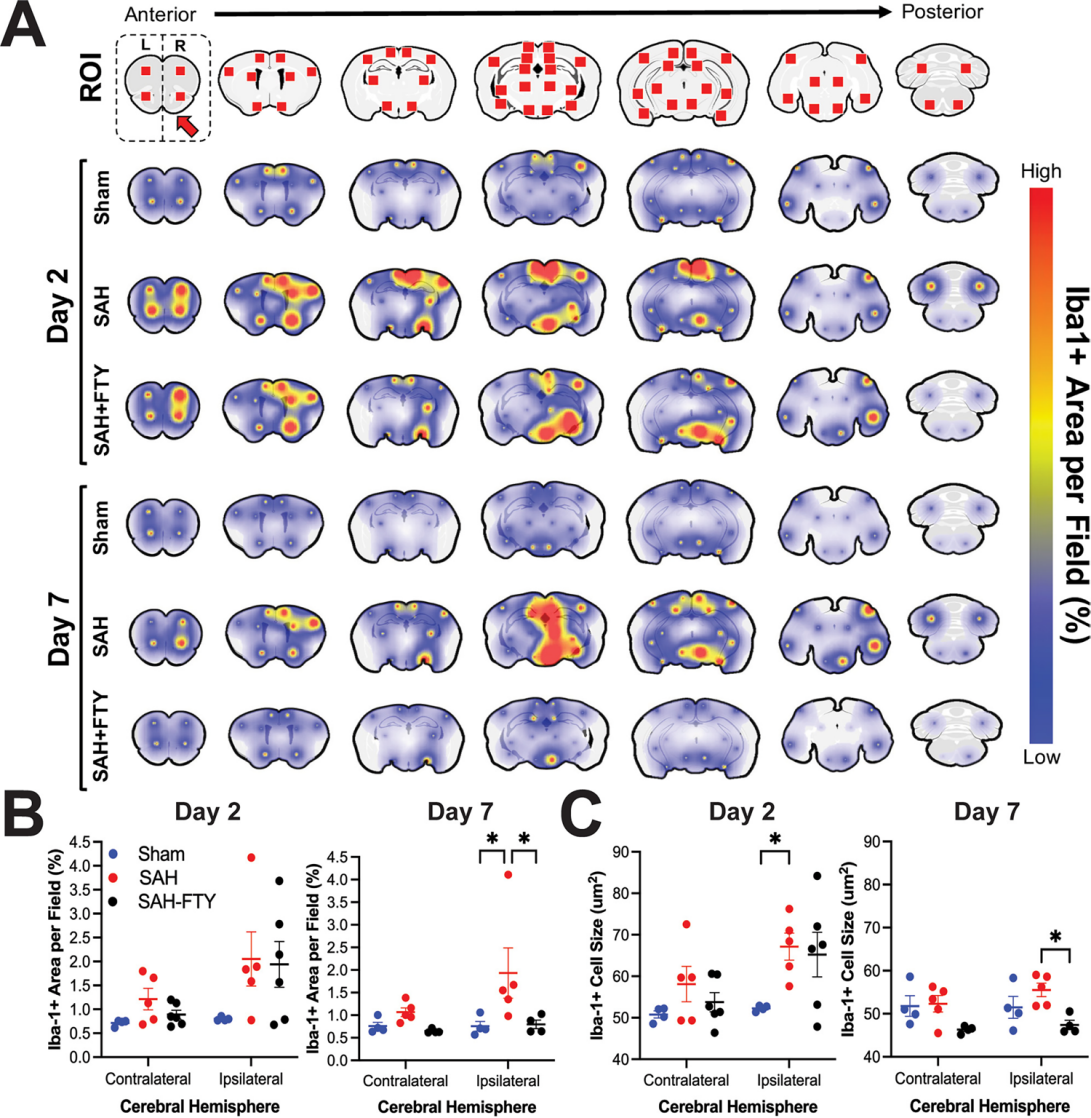
Spatiotemporal mapping reveals increased microglia and cell size that are attenuated by FTY treatment. 64 regions of interest (ROIs) were selected spanning the entire rostral-caudal axis. Iba-1^+^ area per field within each ROI was then quantified and mapped onto the rodent brain in sham, untreated SAH, and SAH-FTY animals at 2- and 7-days following surgery. (**A**) There was a clear association between location of bleeding and Iba-1^+^ cell responses, although several distant sites also showed increased signal, typically ipsilateral to arterial perforation. At 2 days, Iba-1^+^ was increased in both untreated SAH and SAH-FTY animals compared to shams. At 7 days, SAH-FTY animals displayed a drastic reduction in Iba-1^+^ compared to untreated SAH animals. (**B**) Increased Iba-1^+^ was observed in the ipsilateral hemisphere of SAH animals, most pronounced on day 7, where Iba-1^+^ was significantly increased in SAH animals compared to shams and attenuated by FTY. Day 2: Two-way ANOVA with main effects of group (*p* = 0.032), Šídák’s multiple comparisons test for ipsilateral hemisphere: sham vs. SAH *p* = 0.072, SAH vs. SAH-FTY *p* = 0.993, for contralateral hemisphere: sham vs. SAH: *p* = 0.725, SAH vs. SAH-FTY *p* = 0.875. Day 7: Two-way ANOVA with main effects of group (*p* = 0.014), Šídák’s multiple comparisons test for ipsilateral hemisphere: sham vs. SAH *p* = 0.020, SAH vs. SAH-FTY *p* = 0.025, for contralateral hemisphere: sham vs. SAH: *p* = 0.821, SAH vs. SAH-FTY *p* = 0.645). (**C**) Similar results were observed with Iba-1^+^ cell size, where increased cell size was observed at 2 days in SAH animals compared to shams, but by day 7 SAH-FTY animals had reduced Iba-1^+^ cell size compared to untreated SAH animals. Day 2: Two-way ANOVA with main effects of group (*p* = 0.026) and hemisphere (*p* = 0.024), Šídák’s multiple comparisons test for ipsilateral hemisphere: sham vs. SAH *p* = 0.037, SAH vs. SAH-FTY *p* = 0.974, for contralateral hemisphere: sham vs. SAH: *p* = 0.476, SAH vs. SAH-FTY *p* = 0.770. Day 7: Two-way ANOVA with main effects of group (*p* = 0.003), Šídák’s multiple comparisons test for ipsilateral hemisphere: sham vs. SAH *p* = 0.330, SAH vs. SAH-FTY *p* = 0.013, for contralateral hemisphere: sham vs. SAH: *p* = 0.996, SAH vs. SAH-FTY *p* = 0.081. *n* = 4–6 animals per group. Data shown as mean ± standard error.

To assess the full extent of microglial activation in both cortical and subcortical regions for each animal, we quantified microglia in 64 regions throughout the rat brain by measuring Iba-1^+^ area (Fig. 7A). SAH animals showed a marked increase in Iba-1^+^ staining throughout the brain at both 2 and 7 days. The spatial distribution consistently showed higher Iba-1^+^ staining near the SAH at the ventral brain surface. When ROIs on the ipsilateral and contralateral hemisphere were combined, main effects of hemisphere on the percentage of Iba-1^+^ area were observed at 2 days (*p* = 0.032) but not at 7 days (*p* = 0.155). While there was a trend for main effects of group on day 2 (p = 0.070), this was more pronounced by day 7 (*p* = 0.014). Analysis of Iba-1^+^ area within the ipsilateral hemisphere at day 2 suggested increased microglia in SAH animals compared to shams; however, this was not statistically significant (2.052 ± 1.267% vs. 0.799 ± 0.039%, *p* = 0.072; Fig. 7B, left).

By 7 days, there continued to be increased Iba-1 in SAH animals compared to shams (1.932 ± 1.244% vs. 0.755 ± 0.212%, *p* = 0.020). While there were no significant differences in Iba-1 2 days after SAH (*p* = 0.993), treatment with FTY significantly reduced microglia by 7 days ipsilaterally (1.932 ± 1.244% vs. 0.796 ± 0.190%, *p* = 0.025, Fig. 7B, right). No differences between groups were observed within the contralateral hemisphere (*p* > 0.05). All three groups showed reduced Iba-1^+^ area over time between 2 and 7 days (0.04%, 0.12%, and 1.15% decrease for sham, SAH, and SAH-FTY animals, respectively); however, the reduction was most pronounced in SAH-FTY animals. Compared to untreated SAH animals, there was a 9.58-fold higher reduction in Iba-1^+^ microglia in SAH-FTY animals between days 2 and 7.

SAH also increased microglial size, with main effects of group (*p* = 0.026) and hemisphere (*p* = 0.024) observed on day 2 but only main effect of group (*p* = 0.003) observed on day 7. Pairwise analysis of Iba-1^+^ cell size within the ipsilateral hemisphere revealed increased size of Iba-1^+^ cells in SAH animals compared to shams at day 2 (67.136 ± 3.257 µm^2^ vs. 52.271 ± 0.346 µm^2^, *p* = 0.037, Fig. 7C, left). By day 7, FTY treatment of SAH animals reduced cell size (55.527 ± 1.559 µm^2^ vs. 47.393 ± 1.087 µm^2^, *p* = 0.013, Fig. 7C, right). Like Iba-1^+^ area, Iba-1^+^ cell size decreased between 2 and 7 days in all groups (0.79 µm^2^, 11.61 µm^2^, and 17.84 µm^2^ decrease for sham, SAH, and SAH-FTY animals, respectively) but the largest difference over time was observed in SAH-FTY animals.

Microglial activation was also location-specific between SAH and sham animals as well as between SAH and SAH-FTY animals (Supplementary Table 2). On day 2, increased Iba-1^+^ staining was observed within the ipsilateral hypothalamus, retrosplenial cortex, motor cortex, orbitofrontal cortex, somatosensory cortex, and olfactory tubercle/piriform cortex (each *p* < 0.05). The most pronounced increases were observed within the middle hypothalamus (*p* = 0.004), anterior hypothalamus (*p* = 0.002), retrosplenial cortex (*p* = 0.003), and motor cortex (*p* = 0.019). FTY treatment reduced Iba-1^+^ staining on day 2 only at the ipsilateral retrosplenial cortex (*p* = 0.013), motor cortex (*p* = 0.016), and somatosensory cortex (*p* = 0.021). By day 7, there was increased Iba-1^+^ in SAH animals compared to shams in the ipsilateral thalamus, hippocampus, hypothalamus, amygdala, ventral tegmental area, visual cortex, entorhinal cortex, and motor cortex in addition to the contralateral hippocampus (each *p* < 0.05). The most pronounced increases were observed within the anterior thalamus, hippocampus, and hypothalamus (each *p* < 0.001). FTY treatment reduced Iba-1^+^ staining in most of these areas except for the anterior hypothalamus (*p* = 0.081) and contralateral hippocampus (*p* = 0.336). The most pronounced reductions in Iba-1^+^ staining in FTY-treated animals were in the amygdala (*p* = 0.003), anterior thalamus (*p* < 0.001), and middle hypothalamus (*p* < 0.001).

Overall, SAH in this rodent model induces widespread microglial activation through many brain regions, some far removed from the site of hemorrhage. Cortical structures appear to be affected earlier while deeper grey matter structures become affected at later stages. FTY treatment not only reduces microglial activation after SAH, but appears to exhibit its strongest effect by day 7 and in deeper cortical locations.

### Microglia increase phagocytic activity when exposed to blood products

**Figure 8:**
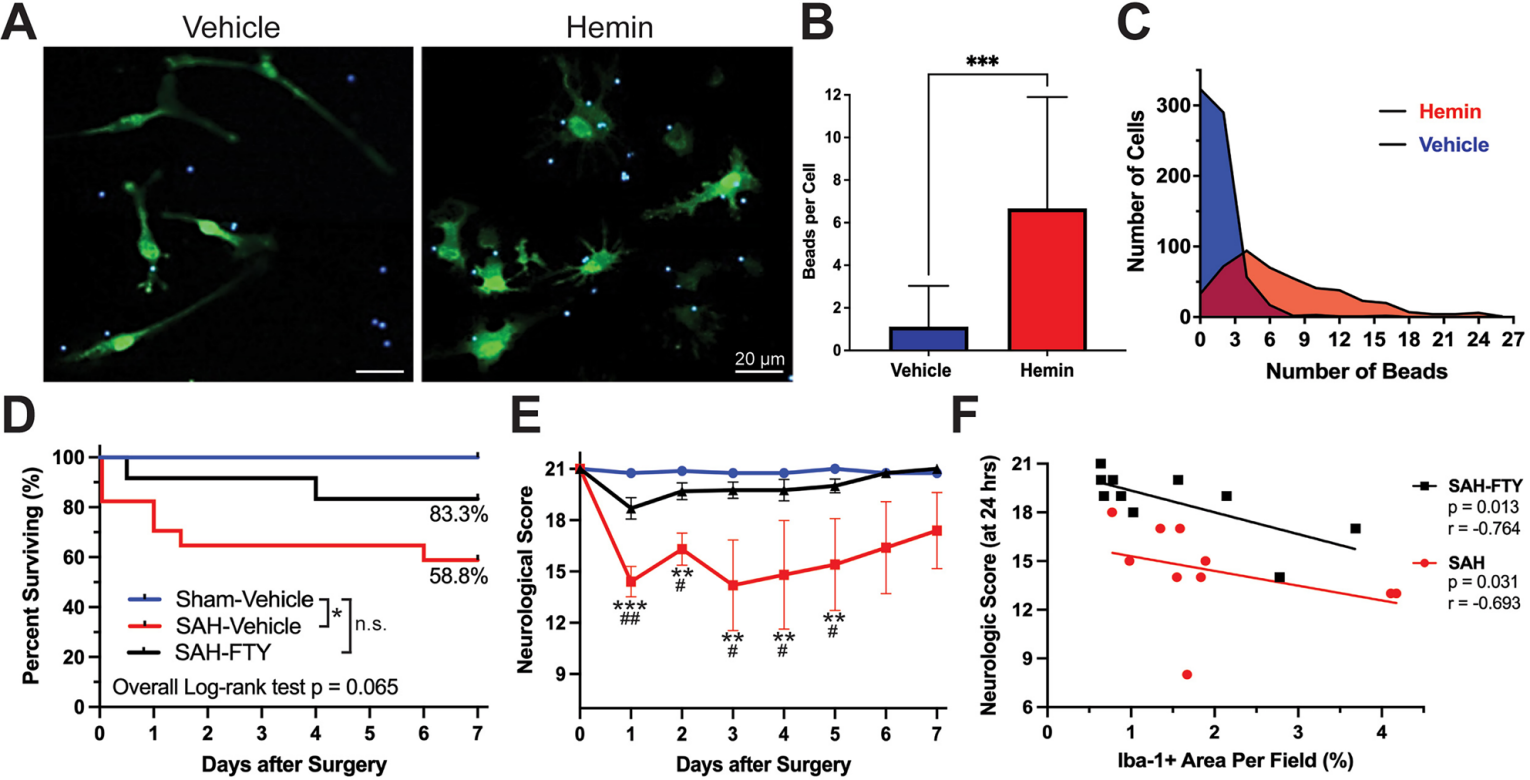
Targeting microglial activation after experimental SAH improves neurologic outcome and survival. (**A**–**C**) Primary CX_3_CR-1^GFP/GFP^ microglia exposed to 40 µM hemin for 12 h in the presence of 2 µm fluorescent blue latex beads demonstrated increased phagocytosis in response to hemin. (**A**) Images of GFP-expressing microglia (green) show increased bead (blue) phagocytosis and morphological changes to a more amoeboid-shape following exposure to hemin, features consistent with microglial activation. (**B**,**C**) There was a significant increase in phagocytic activity of microglia in the presence of hemin (*p* < 0.001, Mann–Whitney test). (**D**) FTY treatment resulted in improved survival in SAH animals up to 7 days after surgery (log-rank (Mantel-Cox) test, *p* = 0.065). Percentages represent the proportion of animals surviving by day 7. (**E**) Neurologic severity score showed deficits in untreated SAH animals that were prevented with FTY treatment. Two-way ANOVA: Time: *p* = 0.007, Group: *p* < 0.001. Šídák’s multiple comparison test: sham vs. SAH *p* < 0.001, sham vs. SAH-FTY *p* = 0.541, SAH vs. SAH-FTY *p* < 0.001. *n* = 8–10 per group. Data shown as mean ± standard error. (**F**) Scatterplot of Iba-1 + area per field (%) versus neurological score 24 h after surgery. Each data point represents one animal. Spearman correlation test. *r* and *p* values are reported. *n* = 10 per group.

Given the strong co-localization of microglia and erythrocyte break down products with Prussian blue (Fig. 6B), we measured the effect of one such blood product (hemin) on primary microglia. CX_3_CR-1^GFP/GFP^ microglia exposed to 40 µM hemin showed increased amoeboid morphology as well as increased phagocytic activity as measured by a latex bead phagocytosis assay compared to vehicle control (Fig. 8A). Hemin-exposed microglia showed a near six-fold increase in phagocytosis, with an average of 6.55 ± 3.91 beads per cell compared to 1.12 ± 0.30 beads per cell in vehicle-treated controls (*p* = 0.032, Fig. 8B). This represented a significant shift in microglial phagocytic activity following exposure to the blood product hemin, even when given at concentrations lower than expected in vivo (Fig. 8C).

### FTY reduces microglial responses after SAH with improved survival and neurological function

We next assessed the impact of SAH with and without FTY treatment on survival and neurologic function. Among a total of 40 rats used in our drug-treatment study, there were 8 sham, 18 SAH, and 14 SAH-FTY animals. Shams had 0% mortality and SAH animals had 41.2% mortality (log-rank test, *p* = 0.042, Fig. 8D). SAH-FTY animals had reduced mortality of 16.7% (Fig. 8D). Overall differences across the three groups approached significance (*p* = 0.065).

Compared to untreated SAH animals, SAH-FTY animals showed a marked improvement in neurologic function (group *p* < 0.001, time *p* = 0.007). This difference was present on days 1 (median neurologic score 14.5 vs 19, *p* = 0.002), 2 (17.5 vs. 20, *p* = 0.021), 3 (16 vs. 19.5, *p* = 0.010), 4 (19 vs. 20, p = 0.026), and 5 (19 vs. 20, p = 0.043), after which SAH animals showed variable rates of neurologic recovery that overlapped with SAH-FTY animals (Fig. 8E). Together, these results suggest that FTY not only reduces pathological microglial activation, but also improves early mortality and neurologic impairment following experimental SAH.

Neurologic function was related to the extent of microglial activation. There was a clear correlation between ipsilateral Iba-1^+^ area and neurological score (Fig. 8F) for both untreated SAH (*p* = 0.031,* r* = − 0.693) and SAH-FTY (*p* = 0.013, *r* = − 0.764) animals regardless of time point. These data suggest that greater microglial responses after SAH drive poor neurologic outcome that can be attenuated by FTY treatment.

## Discussion

SAH has limited treatment options, and historically much attention has been given to targeting cerebral vasospasm and DCI. However, recent studies have demonstrated the impact of EBI on SAH outcome, suggesting that earlier interventions may prevent the development of life-threatening complications. One prominent feature of EBI is a robust inflammatory response, characterized by changes in resident and circulating immune cells that promote a pro-inflammatory environment which may lead to secondary neuronal injury and synaptic rewiring^6^. Disruption to synaptic networks can lead to abnormal neuronal firing patterns, reflected in altered EEG patterns.

In this study, we investigated both acute and chronic sequelae of SAH. We demonstrated the utilization of MRI to confirm the extent and location of subarachnoid bleeding in rats. Both short- and long-term EEG changes correlated with neurobehavioral abnormalities, with SAH animals developing early EEG spectral changes characterized by increased delta slow wave and decreased relative alpha activity. These spectral changes colocalized with areas of SAH and were more pronounced in the ipsilateral cerebral hemisphere. Changes in SPR have been shown to be associated with cognitive function, where a shift in power toward lower frequencies was associated with pathological brain aging in patients with Alzheimer’s disease and type 2 diabetes^32^. Use of continuous EEG in SAH patients has demonstrated acute increases in delta activity with corresponding decreases in RAV and ADR^8–11^. Previous studies, including by Claassen et al. (2004), showed that decreases in ADR can be used in the early detection of DCI in SAH patients when secondary brain injury is still reversible and this has been further supported in recent studies^44–46^. Our findings in SAH rats recapitulate these human observations and demonstrate the ability of our model to facilitate future mechanistic and therapeutic studies. While DCI has been challenging to identify and study in preclinical SAH due to differences in its timing and occurrence between humans and animals^42^, future studies could investigate EEG both for acute ischemic changes and in long-term studies exploring the development of epileptiform activities and cortical spreading depolarizations^47–49^. We did observe interictal spiking and, more rarely, seizures in our animals, warranting longer-term studies since seizures after SAH may take months or years to develop^50^.

In tandem with EEG changes, significant neurobehavioral changes were observed up to 3 months after SAH. Early, large deficits in neurologic function showed some recovery over time, but remained consistently below the level of controls. Additionally, moderate deficits in spontaneous locomotor activity were identified via the open field test. Two other studies have conducted behavioral testing in SAH models; however, they examined a single timepoint and produced mixed results^51,52^. In our longitudinal study, we observed some recovery of locomotor activity during the first week after SAH prior to a subsequent relapse in suppressed locomotion after 3–4 weeks. It is possible that this data reflects acute recovery from the initial SAH injury followed by a delayed manifestation of neurobehavioral deficits resulting from secondary injury.

Our findings demonstrate concurrent changes in EEG activity, neurobehavior, and upregulation of reactive microglia in the rodent brain following SAH. It is known that extravasated red blood cells release a host of bioactive, neurotoxic, and inflammatory molecules that trigger microglial activation^53–56^. Our in vitro results demonstrated that microglia indeed exhibit an activated phenotype following exposure to blood products, as indicated by increased amoeboid morphology and dramatically upregulated phagocytic activity. Within the rat brain, microglia increased in size and number as early as 48 h after SAH and remained significantly elevated for at least 7 days. Previous studies of post-SAH microglial responses in the rodent brain have only focused on single or few select areas of interest often at or near extravasated blood^15,57,58^. However, by quantifying microglial responses throughout the entire brain, we found that microglial activation also increases in cortical and subcortical areas far removed from the injury site. These findings corroborate those described by Zheng et al. (2020), which showed in a mouse EVP model that increased Iba-1^+^ cells were largely found in the cortex adjacent to the perforation site up to 10 days after SAH^58^. Activation of cortical microglia after SAH may underlie synaptic changes that manifest within EEG recordings.

Microglial responses following SAH appear to be dynamic and heterogeneous, with both pro- and anti-inflammatory effects. Some studies have shown that depletion of microglia ameliorates neuronal cell death and vasospasm after SAH^59,60^. In contrast, other studies report beneficial effects of microglia after SAH via heme-oxygenase 1-dependent erythrophagocytosis, although this mechanism may become overwhelmed in SAH^61,62^. It seems that post-SAH microglial activation is a “double-edged sword,” with both neuroprotective and deleterious effects depending on the degree of injury and duration of response. It is likely that the role of microglia is time-dependent, whereby early after hemorrhage, a pro-inflammatory response is beneficial but over time this should shift to an anti-inflammatory, reparative phenotype to prevent secondary neuronal injury.

To understand these and other possible functional roles of activated microglia following SAH, we targeted microglial activation using the immunomodulatory drug FTY. While FTY had minimal effects on microglia 2 days after SAH, by day 7 the number and size of microglia was significantly reduced throughout the brain. Studies in ischemic stroke models have shown that FTY skews microglial responses towards an anti-inflammatory phenotype^63,64^, but whether FTY exerts similar effects in SAH remains unknown. As FTY does not have a single molecular or cellular target, it may have multiple mechanisms of action. Previous work in our lab has shown that FTY reduces peripheral leukocyte adhesion to pial vasculature 2 days after SAH, suggesting peripheral effects of FTY may precede the central effects observed in this study^20^. While further work remains to be done, it is clear that FTY treatment had beneficial effects on survival and neurologic function after SAH.

This study has several limitations. Although it falls within the typical range reported in SAH models^42^, the relatively high mortality rate of SAH animals limited the ability to obtain a larger sample size. However, the longitudinal design of our study with repeated measures allowed sufficient power to detect statistically significant results. Our results likely underestimate the true extent of EEG and neurobehavioral changes as only animals that survived were able to be analyzed. Furthermore, while Iba-1 is the most ubiquitously used microglial marker, it is also expressed by peripheral monocytes which may infiltrate the CNS following SAH. Although several studies have shown that the vast majority of Iba-1^+^ cells in the post-SAH brain are resident microglia^58,65^, future work should employ newer, microglia-specific markers^66^. Given the complex heterogeneity of microglia, there is a need for future studies to employ single-cell RNA sequencing to characterize microglial subpopulations based on differential gene expression. Future studies could also employ novel in vivo techniques such as positron emission tomography using radioactive ligands against CSF1R or 18-kDa translocator protein (TSPO) to image neuroinflammation in the same animals undergoing EEG recordings^67,68^. It will also be essential to perform long-term EEG studies in animals treated with FTY to look directly at the effects of attenuating microglial responses on long-term EEG changes after SAH. This could be done with FTY, an FDA-approved medication currently in clinical use, as well as pre-clinical medications that target microglia more specifically^69^. Finally, our study used male rats aged 2–4 months and future studies should examine sex and age as independent factors that may influence both inflammation and EEG outcomes.

Overall, our results provide evidence that SAH drives diffuse microglial activation in the brain that contributes to short- and long-term impairments, in particular mapping to the same cortical areas where spectral EEG abnormalities are observed. The long-term SAH model we developed here combines MRI, EEG, neurobehavioral testing, and histological studies to investigate how neuroimmune responses drive neurologic impairment. Since many of the EEG changes recapitulate those observed in human patients, this model system is highly translational to patients and will therefore aid in developing therapeutic strategies such as FTY to reduce early inflammation and improve patient outcomes after SAH.

### Supplementary Information

Supplementary Information 1.

Supplementary Figure 1.

Supplementary Figure 2.

Supplementary Figure 3.

Supplementary Figure 4.

Supplementary Table 1.

Supplementary Table 2.

## Data Availability

Data is provided within the manuscript and supplementary information files. The datasets generated during and/or analyzed during the current study are otherwise available from the corresponding author on reasonable request.
