# Supplementary material for: Diffuse microglial responses and persistent EEG changes correlate with poor neurological outcome in a model of subarachnoid hemorrhage
Source: Sci Rep. 2024 Jun 13;14:13618. doi: 10.1038/s41598-024-64631-2 (PMC11176397; doi:10.1038/s41598-024-64631-2)
Supplement: Supplementary file 1 — Supplementary Information 1. [file 41598_2024_64631_MOESM1_ESM.docx]

**Supplementary Information**

**Supplementary Figure 1. Widespread reduction in EEG relative alpha variability after experimental SAH. A)** Spectral map of relative alpha variability (RAV = *α* / total) displayed across all six electrodes shown for sham and SAH over time. **B)** Quantitative EEG analysis revealed widespread reduction in RAV in the first 14 days after SAH across all electrodes. Three-way ANOVA with main effects of time, group, electrode (each *p*<0.001) and interactions between group and electrode (*p*=0.038) and group and time (*p*<0.001). No interaction was detected between electrode and time (*p*>0.999) or between all three variables (*p*>0.999). Within each electrode, Šidák’s multiple comparisons test was used to assess for between-group differences at each time point. *n*=6-10 animals per group. Data shown as mean ± standard error.

**Supplementary Figure 2. Decreased EEG alpha-delta ratio in SAH animals is most pronounced at distant anterior electrodes. A)** Spectral map of alpha-delta ratio (ADR = *α / δ*) across all six electrodes shown for sham and SAH groups over time. **B)** Quantitative EEG analysis revealed moderate reduction in ADR in the first 14 days after SAH across all electrodes, but most prominent in the anterior electrodes (L1, R1, R2). Three-way ANOVA with main effects of time, group, and electrode (each *p*<0.001) and interactions between group and electrode (*p*=0.028) and group and time (*p*<0.001). No interaction was detected between electrode and time or between all three variables (both *p*>0.999). Within each electrode, Šidák’s multiple comparisons test was used to assess for between-group differences at each time point. *n*=6-10 animals per group. Data shown as mean ± standard error.

**Supplementary Figure 3. Additional correlations between neurobehavioral changes and quantitative EEG parameters following experimental SAH. A-B)** Overall, SAH animals showed **(A)** reduced vertical counts or rearing compared to shams (main effect of group, *p*=0.026); **(B)** however, there was no difference in the ratio of counter-clockwise to clockwise (CCW:CW) turns (main effect of group, *p*=0.328). *n*=6-10 animals pr group. Data shown as median ± interquartile range. **C-D)** Significant positive correlations between neurological score and both the (**C**) relative alpha variability (RAV, *p*<0.001, *r*=0.462) and (**D**) alpha-delta ratio (ADR, *p*<0.001, *r*=0.475). **E-J)** Correlations between open field test and quantitative EEG. There were no significant correlations between ambulatory distance and any of the quantitative EEG variables, including **(E)** spectral power ratio (SPR, *p*=0.913, *r*=0.005), **(F)** mean delta power (*p*=0.103, *r*=0.071), **(G**) RAV (*p*=0.321, *r*=0.045), or **(H)** ADR (*p*=0.702, *r*=-0.017). Similarly, there was no correlation between percentage of time spent in the center of the open field and either **(I)** SPR (*p*=0.355, *r*=0.041) or (**J**) mean delta power (*p*=0.272, *r*=-0.048). Spearman correlation with individual data points shown overlaid with a linear regression line and 95% confidence interval.

**Supplementary Figure 4. SAH animals treated with FTY display a similar extent of SAH as those without FTY but had improved neurological function. A)** Regional cerebral blood flow showed successful induction of SAH with no differences between untreated SAH and SAH-FTY animals. Two-way ANOVA with main effects of group and time (both *p*<0.001) and no interaction (*p*=0.087). Šidák’s multiple comparisons test comparing untreated SAH and SAH-FTY animals to shams. Significance denoted as *Sham vs. SAH; ^#^Sham vs. SAH-FTY. **B)** *V_hypo_* or volume of quantified blood was similar between SAH and SAH-FTY animals. One-way ANOVA (*p*=0.002) with Šidák’s multiple comparisons test: sham vs. SAH *p*=0.002, SAH vs. SAH-FTY *p*=0.803, sham vs. SAH-FTY *p*=0.013. **C)** No differences in total brain volume were observed (*p*>0.05). **D)** Ventricular size was enlarged in SAH animals compared to shams; however, this did not meet our pre-defined threshold for statistical significance (*p*=0.053). No difference between sham vs. SAH-FTY (*p*=0.312) or SAH vs. SAH-FTY (*p*=0.691) was observed. *n*=8-10 per group. Data shown as mean ± standard deviation.

**Table 1. Spectral changes in quantitative EEG absolute and relative frequency bands between sham and SAH animals.**

**Table 2. Spatiotemporal microglial responses at specific neuroanatomical locations after experimental SAH.**

**Supplementary Methods**

**Endovascular Perforation Model**

Rats were anesthetized with isoflurane in 100% oxygen. Analgesia was provided by local injection of 0.2 mL 1% lidocaine and 0.3 mL 0.0125% bupivacaine at incision sites in addition to perioperative subcutaneous 0.1 mg/kg buprenorphine. Prior to surgery, animals were intubated with an endotracheal tube and placed on a ventilator (TOPO Small Animal Ventilator, Kent Scientific Corporation). Ventilator settings were as follows: peak inspiratory pressure 13 cm H_2_O, respiratory rate 58 beats per minute, and flow 2 L/min. Body temperature was maintained at 37^o^C for the duration of the procedure using a rectal probe and heating pad.

A 3-cm incision was made over the midline of the scalp. Skin and the temporalis muscle were retracted, and fascia was dissected. The underlying skull was cleaned and dried. Regional cerebral blood flow (rCBF) was continuously monitored before, during, and after surgery up to 30 minutes by Laser Doppler flowmetry (Perimed Inc.). The Doppler probe was attached to the skull over the territory of the right middle cerebral artery (MCA, 1 mm posterior to bregma, 3 mm right of midline). Once stable rCBF recordings are achieved, the animal was carefully rotated to the supine position.

A 3.5-cm midline incision was made in the anterior neck. Glandular tissue and fascia were exposed and dissected. The underlying muscles of the anterior neck were exposed, and the right common carotid artery (CCA) was identified. The vessel was carefully dissected anteriorly from the surrounding adventitia, smaller blood vessels, and nerves, with particular attention paid to avoiding damage to the adjacent vagus nerve. The dissection was performed anteriorly until the bifurcation of the CCA, at which point the external carotid artery (ECA), internal carotid artery (ICA), superior thyroid artery (STA), and pterygopalatine artery were dissected. The STA and pterygopalatine artery were isolated, cauterized with bipolar cautery, and divided to prevent blood loss. The ECA was dissected anteriorly, cauterized, and divided to create a proximal ECA stump that was retracted to align with the ICA. The CCA and ICA were then temporarily occluded with aneurysm clips, and a small arteriotomy was made in the ECA. A hollow polytetrafluoroethylene (PTFE) tube (SUBL-080, OD 0.2 mm, ID 0.1 mm, Braintree Scientific, Inc.) containing a tungsten wire (W91, 0.076 mm diameter, Scientific Instrument Services, Inc.) was inserted into the ECA, secured with a silk suture, and the ICA aneurysm clip was removed. The tubing was then carefully advanced distally approximately 2 cm until a drop in rCBF occurs, at which point the tubing reached the bifurcation of the ICA and temporarily occluded blood flow to the right MCA. At this point, the tungsten wire was fully advanced approximately 2 mm within the tubing, puncturing the ICA bifurcation and establishing a hemorrhagic lesion (Fig. 1A). The tungsten wire was retracted, followed by the PTFE tubing. The ICA aneurysm clip was temporarily replaced and the ECA arteriotomy was cauterized and closed. Total occlusion time was less than five minutes. The anterior neck incision was closed and rCBF is monitored and recorded for 30 minutes. Sham animals received the identical procedure except for advancement of the tungsten filament, and therefore did not experience a hemorrhagic lesion.

Following the procedure, sham and SAH animals received 2.5 mL of warm 0.9% sterile saline intraperitoneally and topical triple antibiotic ointment (neomycin, polymyxin B, and bacitracin) applied to incision sites. Rats were placed into a recovery chamber maintained at 37^o^C for one hour before being returned to their home cages with access to food and water ad libitum. The rats are observed daily over for 1-week post-surgery to ensure that they receive adequate hydration and nutrition. Rats were weighed daily. Any animal that appeared moribund (i.e., unable to move, ambulate, drink, or eat, or otherwise unresponsive to external stimuli) was determined to meet humane endpoint criteria and euthanized. After the first week, animals were monitored at minimum twice weekly for the remainder of the study.

**MRI Image Acquisition**

Animals were imaged in an Agilent 9.4 Tesla MR scanner (Agilent Technologies) with a 72-mm ID quadrature birdcage radiofrequency coil (RAPID Biomed) for transmission and a 4-channel surface head coil assembly (RAPID Biomed) optimized for imaging rats as the receiver. Animals were anesthetized with isoflurane via a nose cone. T2-weighted imaging was performed in the coronal plane using a fast spin echo pulse sequence with the following settings: TR = 5000 ms, TE = 60 ms, field of view (FOV) = 40 x 40 mm, averages = 4, matrix size = 256 x 256, slice thickness = 0.75 mm, and inter-slice gap = 0 mm. Total scan time was 10 min 50 sec per animal. DICOM images were analyzed using custom Matlab scripts and Fiji open-source image analysis software (Version 2.0.0-rc-65/1.52p).

**EEG Electrode Implantation**

While anesthetized, rats were placed in a Kopf stereotactic frame for immobilization. The skull was exposed and a 1-mm tip Dremel drill was used to drill seven epidural holes in the skull. Holes were drilled bilaterally in the following locations based on stereotactic coordinates: AP +4 mm (L1 and R1), -1 mm (L2 and R2), and -6 mm (L3 and R3), each lateral 3.5 mm and all relative to bregma. A final hole was drilled over the nasal sinus (10.5 mm anterior, 0.5 mm lateral to the left, relative to bregma) to serve as the reference electrode site. Stainless-steel screw electrodes (PlasticsOne) were then fixed into each hole and connected to plastic pedestals (PlasticsOne). The skull was then dried, and the entire apparatus was fixed with dental acrylic. The ground electrode was incorporated into the headgear itself. Because EEG implants could be disrupted when housed in groups, all rats involved in this study were single housed. Upon arrival to the laboratory, animals were allowed to acclimate for 3 days and then separated into individual cages, then given a minimum of 4 days further for acclimatization.

**Open Field Test**

Testing was performed using a 17-inch x 17-inch square open field activity system (MED-OFA-RS, Med Associates, Inc.). All animals were allowed to habituate to their home cage following delivery for a minimum of three days prior to behavioral testing. Animals were acclimated to the testing room in their home cages for 30 minutes prior to testing. Each recording was conducted for 15 minutes. Time spent in a 5-inch x 5-inch zone in the center of the apparatus was compared to time spent in the periphery.

**Mixed Glial Cell Culture and Microglial Isolation**

Brains from CX_3_CR-1^GFP/GFP^ C57BL/6J mouse pups (post-natal day 0-5, male and female) were dissected under a stereomicroscope into ice-cold Hank’s balanced salt solution (HBSS). The cerebellum, meninges, and choroid were dissected out, the brains were washed in cold HBSS and incubated with 0.25% trypsin and 50 µg DNase I at room temperature. The solution was then washed with 10 mL of complete Dulbecco’s Modified Eagle Medium (DMEM, containing 4.5 g glucose, L-glutamine, 110 mg/L sodium pyruvate, 10% fetal bovine serum [FBS], and 1% antibiotic-antimycotic solution, Gibco® 12430-054). The suspension was then filtered through a 70 µm nylon cell strainer, spun at 300g for 10 min, and subsequently plated in 15 mL complete DMEM and cultured at 37^o^C with 5% CO2. After 24 hours, media was replaced with 15 mL of fresh complete DMEM, and after that two-thirds of the media was replaced every 3-4 days until confluent. Approximately 3-4 brains were cultured per flask. Mixed glial cultures became confluent 10-12 days after plating. Microglia were then isolated from the mixed glial population by sealing the flasks and shaking at 300 rpm for 30 minutes. The media containing microglia was collected and used for subsequent experiments.

**Fluorescent Bead Phagocytosis Assay**

Cells were plated at 20,000 cells/cm^2^ on 8-well glass chamber slides which were pre-coated with 10 µg/mL poly-L-lysine. Cells were incubated in 300 µL complete DMEM for 24 hours prior to experimentation. The next day, complete DMEM was replaced with FBS-free DMEM and a 2-hour serum starvation was performed. Serum-free media was used for the remainder of the experiment given that serum exposure promotes a non-physiological environment and can induce a reactive, amoeboid state in microglia. Two-micron, fluorescent blue, amine-modified polystyrene latex beads in aqueous suspension (#L0280, Sigma-Aldrich, Inc.) were first incubated in a 1:5 dilution of heat-inactivated FBS prior to experimentation. A stock solution of 50 mM hemin was prepared in 0.25 M sodium hydroxide (NaOH), aliquoted and stored at 4^o^C alongside a vehicle stock solution containing only 0.25 M NaOH. The hemin and vehicle solutions were subsequently diluted in serum-free DMEM to a final concentration of 40 µM, pH was titrated to 7.4, filtered, and applied to cells at the same time as the latex bead solution. Final bead concentration was 0.02% (1:5,000 dilution). Final concentration of vehicle was 133 µM NaOH. Cells were incubated for 12 hours at 37^o^C in 5% CO2, washed, and then fixed in 4% PFA for 30 min. Internalization of beads within microglia was confirmed by confocal z-stack images taken using a Zeiss Laser Scanning Microscope 710 confocal microscope with a Zeiss AxioImager.
