# Supplementary figures and images for: Diffuse microglial responses and persistent EEG changes correlate with poor neurological outcome in a model of subarachnoid hemorrhage
Source: Sci Rep. 2024 Jun 13;14:13618. doi: 10.1038/s41598-024-64631-2 (PMC11176397; doi:10.1038/s41598-024-64631-2)

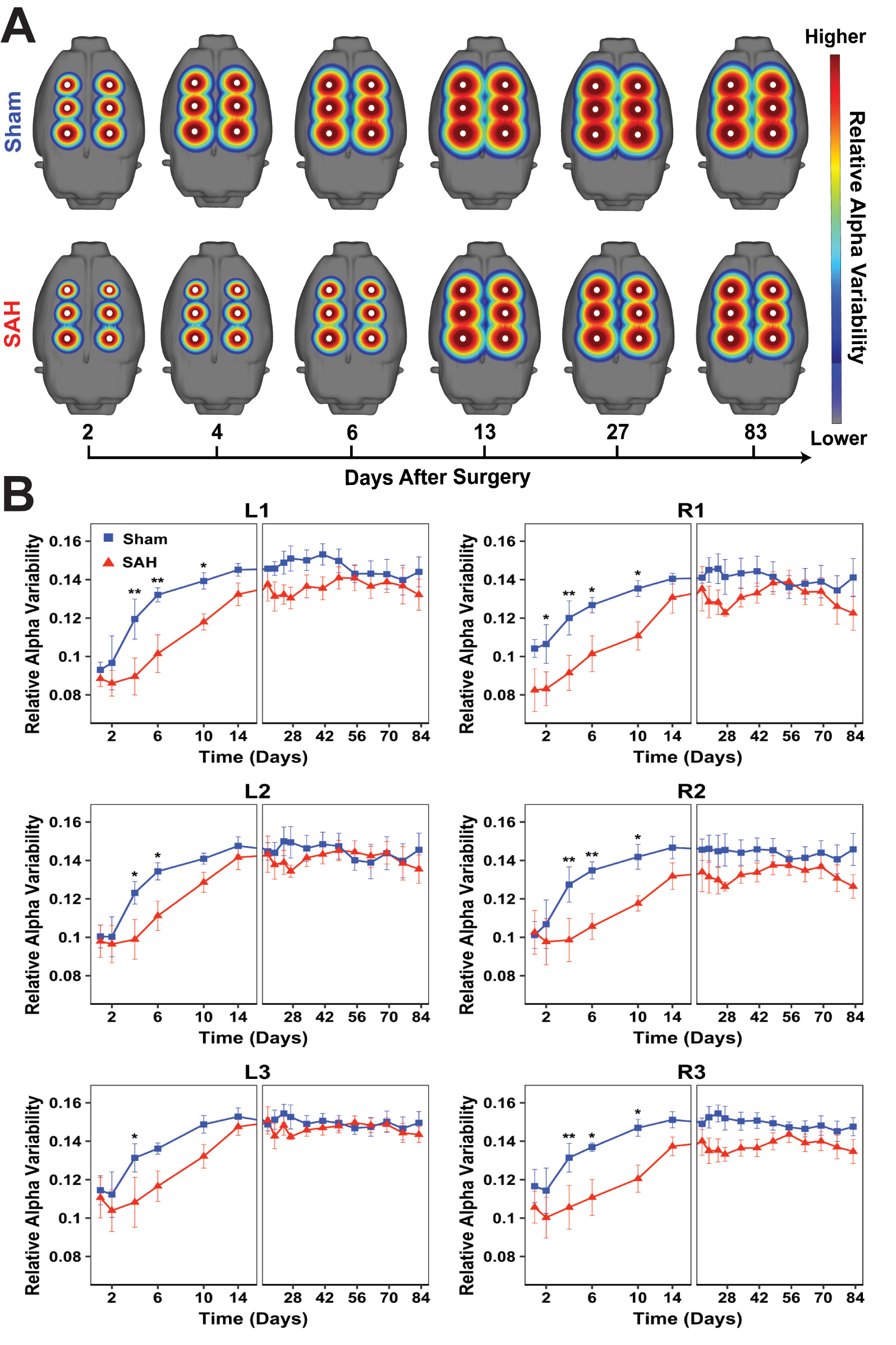

Supplement: Supplementary file 2 — Supplementary Figure 1. [file 41598_2024_64631_MOESM2_ESM.jpg]

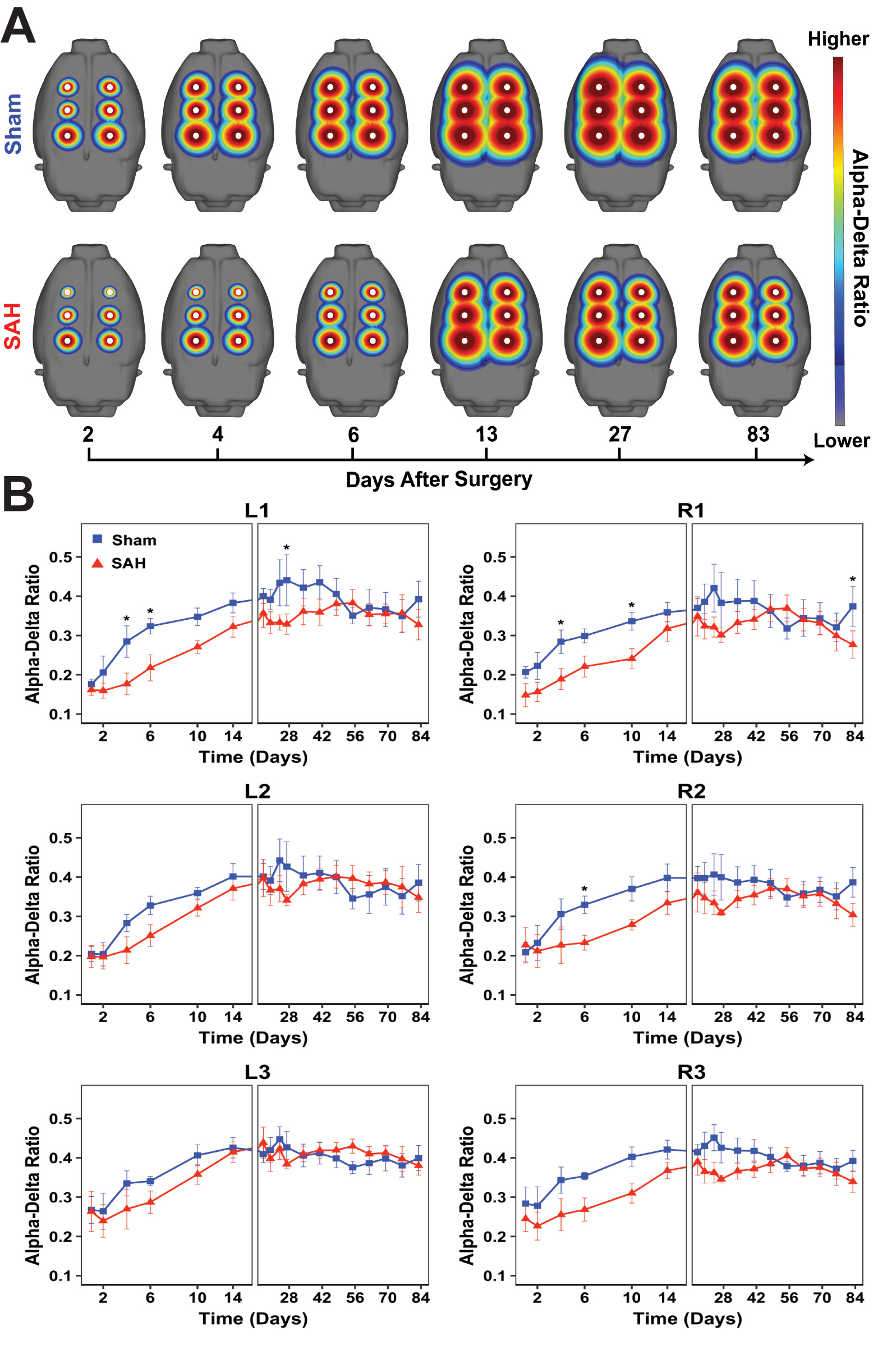

Supplement: Supplementary file 3 — Supplementary Figure 2. [file 41598_2024_64631_MOESM3_ESM.jpg]

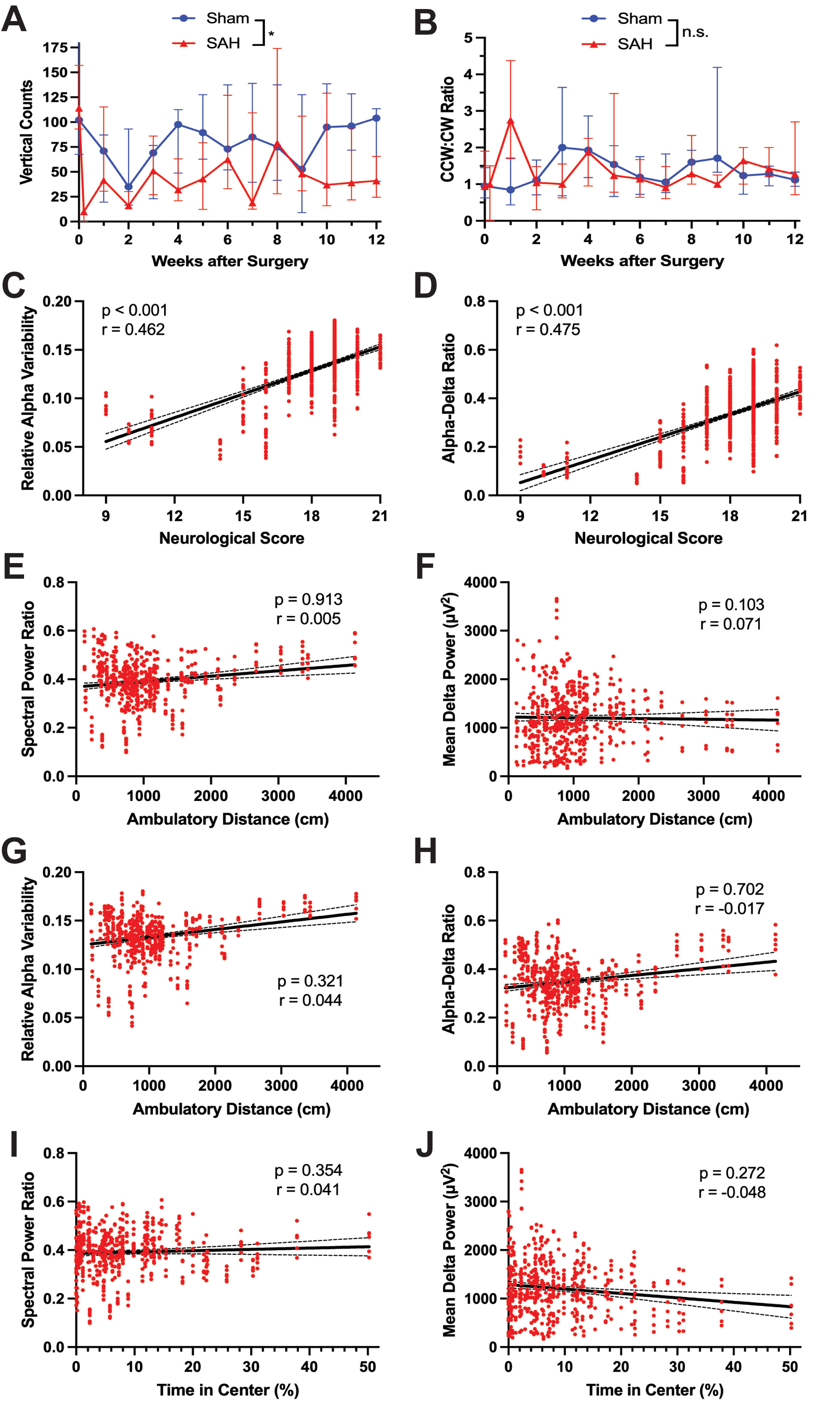

Supplement: Supplementary file 4 — Supplementary Figure 3. [file 41598_2024_64631_MOESM4_ESM.jpg]

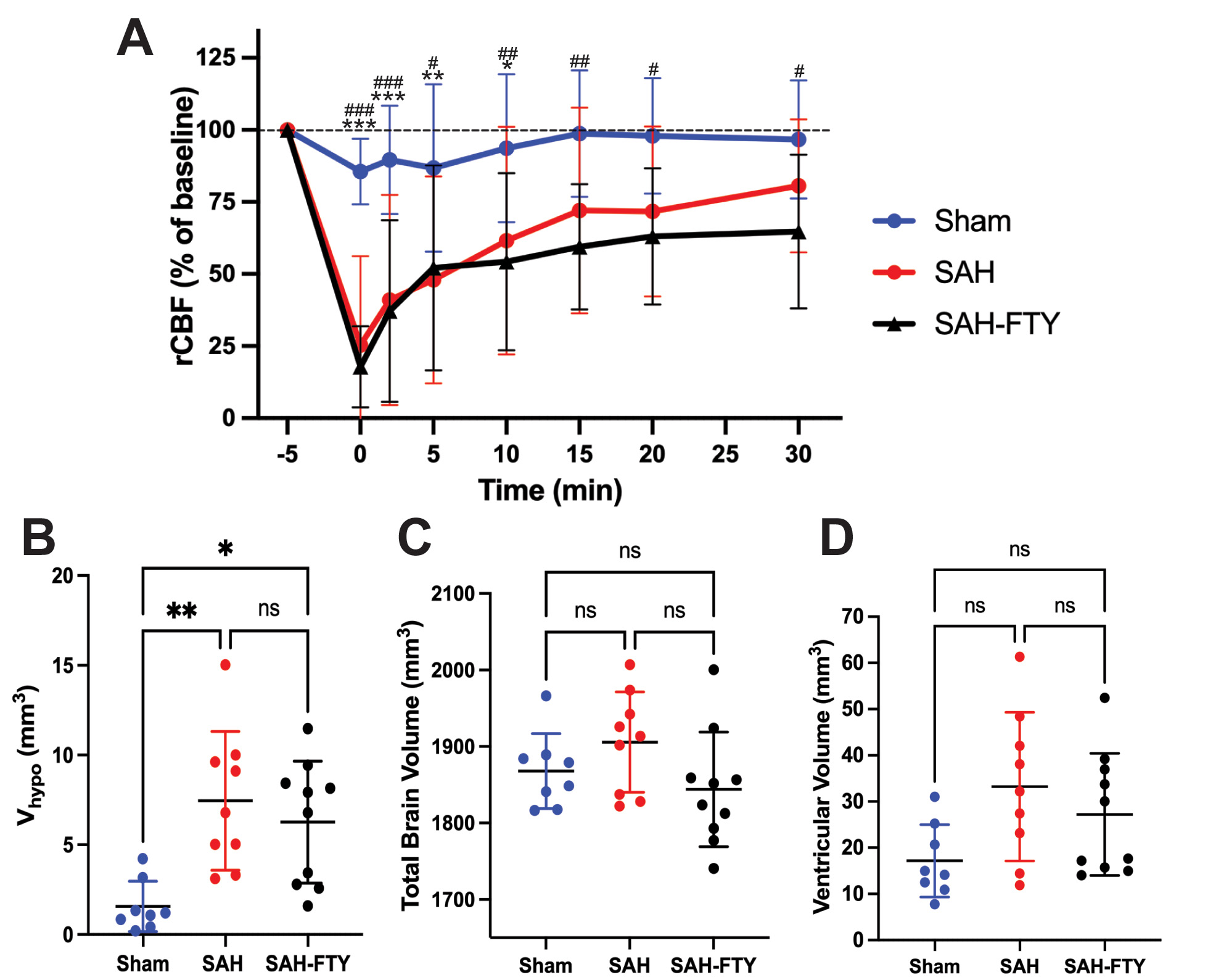

Supplement: Supplementary file 5 — Supplementary Figure 4. [file 41598_2024_64631_MOESM5_ESM.jpg]
