# Supplementary material for: Diffuse microglial responses and persistent EEG changes correlate with poor neurological outcome in a model of subarachnoid hemorrhage
Source: Sci Rep. 2024 Jun 13;14:13618. doi: 10.1038/s41598-024-64631-2 (PMC11176397; doi:10.1038/s41598-024-64631-2)
Supplement: Supplementary file 6 — Supplementary Table 1. [file 41598_2024_64631_MOESM6_ESM.docx]

**Supplementary Table 1. Changes in quantitative EEG absolute and relative frequency bands between sham and SAH animals.**

| **Electrode** | **Absolute Frequency Bands** | | | | **Relative Frequency Bands** | | | |
| --- | --- | --- | --- | --- | --- | --- | --- | --- |
|  | **EEG Parameter** | **Definition** | **Group Difference** | **Days with Significant Differences** | **EEG Parameter** | **Definition** | **Group Difference** | **Days with Significant Differences** |
| L1 | Delta (δ) | 1-4 Hz | No | N/A | RDV | δ/Total | **Yes** | **4, 6** |
|  | Theta (θ) | 4-8 Hz | No | N/A | RTV | θ/Total | **Yes** | **4** |
|  | Alpha (α) | 8-12 Hz | No | N/A | RAV | α/Total | **Yes** | **4, 6, 10** |
|  | Beta (β) | 12-30 Hz | No | N/A | RBV | β/Total | **Yes** | **4, 6, 10, 41** |
|  | Broadband | 1-20 Hz | No | N/A | ADR | α/δ | **Yes** | **4, 6, 27** |
|  |  |  |  |  | SPR | (α+β)/(δ+θ) | **Yes** | **4, 6, 10, 27, 41** |
| **L2** | Delta (δ) | 1-4 Hz | No | N/A | RDV | δ/Total | No | N/A |
|  | Theta (θ) | 4-8 Hz | No | N/A | RTV | θ/Total | No | N/A |
|  | Alpha (α) | 8-12 Hz | No | N/A | RAV | α/Total | **Yes** | **4, 6** |
|  | Beta (β) | 12-30 Hz | No | N/A | RBV | β/Total | **Yes** | **4, 6, 10** |
|  | Broadband | 1-20 Hz | No | N/A | ADR | α/δ | No | N/A |
|  |  |  |  |  | SPR | (α+β)/(δ+θ) | **Yes** | **4, 6, 10** |
| **L3** | Delta (δ) | 1-4 Hz | **Yes** | **4** | RDV | δ/Total | **Yes** | **4, 6** |
|  | Theta (θ) | 4-8 Hz | **Yes** | **55, 69** | RTV | θ/Total | **Yes** | **55** |
|  | Alpha (α) | 8-12 Hz | No | N/A | RAV | α/Total | **Yes** | **4** |
|  | Beta (β) | 12-30 Hz | No | N/A | RBV | β/Total | **Yes** | **4** |
|  | Broadband | 1-20 Hz | No | N/A | ADR | α/δ | No | N/A |
|  |  |  |  |  | SPR | (α+β)/(δ+θ) | **Yes** | **4** |
| **R1** | Delta (δ) | 1-4 Hz | No | N/A | RDV | δ/Total | **Yes** | **2, 4, 6** |
|  | Theta (θ) | 4-8 Hz | No | N/A | RTV | θ/Total | No | N/A |
|  | Alpha (α) | 8-12 Hz | No | N/A | RAV | α/Total | **Yes** | **2, 4, 6, 10** |
|  | Beta (β) | 12-30 Hz | No | N/A | RBV | β/Total | **Yes** | **2, 4, 6, 10, 20** |
|  | Broadband | 1-20 Hz | No | N/A | ADR | α/δ | **Yes** | **4, 10, 83** |
|  |  |  |  |  | SPR | (α+β)/(δ+θ) | **Yes** | **2, 4, 6, 10, 20, 27** |
| **R2** | Delta (δ) | 1-4 Hz | **Yes** | **4** | RDV | δ/Total | **Yes** | **4, 6** |
|  | Theta (θ) | 4-8 Hz | No | N/A | RTV | θ/Total | No | N/A |
|  | Alpha (α) | 8-12 Hz | No | N/A | RAV | α/Total | **Yes** | **4, 6, 10** |
|  | Beta (β) | 12-30 Hz | **Yes** | **10, 20, 24** | RBV | β/Total | **Yes** | **4, 6, 10, 13, 17, 20, 27** |
|  | Broadband | 1-20 Hz | No | N/A | ADR | α/δ | **Yes** | **6** |
|  |  |  |  |  | SPR | (α+β)/(δ+θ) | **Yes** | **4, 6, 10, 13, 27** |
| **R3** | Delta (δ) | 1-4 Hz | **Yes** | **1, 4** | RDV | δ/Total | **Yes** | **4** |
|  | Theta (θ) | 4-8 Hz | **Yes** | **1,55** | RTV | θ/Total | No | N/A |
|  | Alpha (α) | 8-12 Hz | **Yes** | **55** | RAV | α/Total | **Yes** | **4, 6, 10** |
|  | Beta (β) | 12-30 Hz | No | N/A | RBV | β/Total | **Yes** | **4, 6, 10** |
|  | Broadband | 1-20 Hz | **Yes** | **1, 55** | ADR | α/δ | No | N/A |
|  |  |  |  |  | SPR | (α+β)/(δ+θ) | **Yes** | **4, 6, 10** |

RDV: Relative delta variability; RTV: Relative theta variability; RAV: Relative alpha variability; RBV: Relative beta variability; ADR: Alpha-delta ratio; SPR: Spectral power ratio

*Boldface type indicates statistically significant values
