# Supplementary material for: Diffuse microglial responses and persistent EEG changes correlate with poor neurological outcome in a model of subarachnoid hemorrhage
Source: Sci Rep. 2024 Jun 13;14:13618. doi: 10.1038/s41598-024-64631-2 (PMC11176397; doi:10.1038/s41598-024-64631-2)
Supplement: Supplementary file 7 — Supplementary Table 2. [file 41598_2024_64631_MOESM7_ESM.docx]

| **Hemisphere** | **Anatomical Location** | **Sham vs. SAH** | | **SAH vs. SAH-FTY** | |
| --- | --- | --- | --- | --- | --- |
|  |  | **Fold Increase** | **p-value** | **Fold Decrease** | **p-value** |
| **Day 2** | | | | | |
| Ipsilateral | Hypothalamus (Middle) | 5.0610 | **0.0043** | 0.9516 | 0.9728 |
| Ipsilateral | Hypothalamus (Anterior) | 4.5455 | **0.0017** | 1.2029 | 0.6858 |
| Ipsilateral | Retrosplenial Cortex | 4.1368 | **0.0033** | 2.4699 | **0.0130** |
| Ipsilateral | Motor Cortex (Medial Posterior) | 3.6371 | **0.0192** | 1.9845 | 0.1015 |
| Ipsilateral | Orbitofrontal/Motor Cortex | 3.5908 | **0.0342** | 0.9404 | 0.9682 |
| Ipsilateral | Motor Cortex (Medial Middle) | 3.4274 | **0.0074** | 2.4171 | **0.0158** |
| Ipsilateral | Somatosensory Cortex (Anterior) | 3.0983 | **0.0215** | 2.5815 | **0.0211** |
| Ipsilateral | Olfactory Tubercle/Piriform Cortex | 2.8333 | **0.0190** | 1.0452 | 0.9782 |
| **Day 7** | | | | | |
| Ipsilateral | Thalamus (Anterior) | 6.5419 | **0.0003** | 8.1774 | **0.0002** |
| Ipsilateral | Hippocampus (Anterior) | 5.8156 | **0.0003** | 3.1673 | **0.0040** |
| Ipsilateral | Hypothalamus (Anterior) | 4.2977 | **0.0030** | 2.0014 | 0.0811 |
| Contralateral | Hippocampus (Anterior) | 3.9071 | **0.0441** | 1.7786 | 0.3363 |
| Ipsilateral | Hypothalamus (Middle) | 3.7998 | **0.0001** | 4.7646 | **0.0001** |
| Ipsilateral | Amygdala | 3.7936 | **0.0171** | 8.7053 | **0.0029** |
| Ipsilateral | Visual Cortex (Posterior) | 3.7192 | **0.0043** | 3.1636 | **0.0084** |
| Ipsilateral | Entorhinal Cortex | 2.8606 | **0.0226** | 2.8014 | **0.0245** |
| Ipsilateral | Motor Cortex (Lateral Anterior) | 2.5780 | **0.0404** | 3.1677 | **0.0184** |
| Ipsilateral | Ventral Tegmental Area | 2.3449 | **0.0288** | 2.8985 | **0.0100** |

**Supplementary Table 2. Spatiotemporal microglial responses at specific neuroanatomical locations after experimental SAH.**

*Boldface type indicates statistically significant values
